# A Pro-Cathepsin L Mutant Is a Luminal Substrate for Endoplasmic-Reticulum-Associated Degradation in *C. elegans*

**DOI:** 10.1371/journal.pone.0040145

**Published:** 2012-07-02

**Authors:** Mark T. Miedel, Nathan J. Graf, Kate E. Stephen, Olivia S. Long, Stephen C. Pak, David H. Perlmutter, Gary A. Silverman, Cliff J. Luke

**Affiliations:** Department of Pediatrics, Cell Biology and Physiology, University of Pittsburgh School of Medicine, Children's Hospital of Pittsburgh of UPMC and Magee-Womens Hospital of UPMC, Pittsburgh, Pennsylvania, United States of America; University of Pennsylvannia, United States of America

## Abstract

Endoplasmic-reticulum associated degradation (ERAD) is a major cellular misfolded protein disposal pathway that is well conserved from yeast to mammals. In yeast, a mutant of carboxypeptidase Y (CPY*) was found to be a luminal ER substrate and has served as a useful marker to help identify modifiers of the ERAD pathway. Due to its ease of genetic manipulation and the ability to conduct a genome wide screen for modifiers of molecular pathways, *C. elegans* has become one of the preferred metazoans for studying cell biological processes, such as ERAD. However, a marker of ERAD activity comparable to CPY* has not been developed for this model system. We describe a mutant of pro-cathepsin L fused to YFP that no longer targets to the lysosome, but is efficiently eliminated by the ERAD pathway. Using this mutant pro-cathepsin L, we found that components of the mammalian ERAD system that participate in the degradation of ER luminal substrates were conserved in *C. elegans*. This transgenic line will facilitate high-throughput genetic or pharmacological screens for ERAD modifiers using widefield epifluorescence microscopy.

## Introduction

Biological pathways governing protein transcription, synthesis, folding, modification, trafficking and degradation maintain cellular protein homeostasis (proteostasis) [1]. Protein degradative pathways are particularly important, as they are the definitive step for removing toxic accumulations of misfolded or aggregated proteins generated by mutations or environmental stressors. Failure to eliminate these proteins can trigger cellular dysfunction or death that is characteristic of several neurodegenerative disorders, the serpinopathies and some inborn errors of metabolism [1]–[3]. Soluble or oligomeric misfolded proteins in the ER are degraded primarily through a multi-step process, ER-associated-degradation (ERAD; reviewed in [4]). Depending on whether the misfolded proteins reside in the ER lumen or membrane, different sensors recognize the aberrant protein structures [4], [5] and retro-translocate them from the ER to the cytoplasm, where they are ubiquitinated and recognized by the proteasome for degradation [6]–[8]. Studies in yeast have been instrumental in delineating the mechanisms and molecular machinery involved in the turnover of luminal ERAD substrates, with the best characterized example being a mutated (G255R) version of the yeast vacuolar protease, carboxypeptidase Y (CPY*) [9]–[11]. While many of the molecular components of yeast ERAD are conserved in metazoans, significant differences exist [10]–[12]. These differences have prompted the examination of ERAD in multiple model systems including *C. elegans* [13]. However, the *C. elegans* system has not been fully exploited due to the absence of well-defined luminal substrates that permit the visual, biochemical or genetic assessment of putative ERAD modifier genes.

Thus, the purpose of this study was to generate a fluorescent luminal ERAD substrate using a *C. elegans* specific protein. In mammalian systems, mutations in the prepro-region of lysosomal papain-like cysteine peptidases induce protein misfolding and convert them to luminal ERAD substrates that are efficiently degraded by the ubiquitin-proteasome system (UPS) [14]. In *C. elegans*, the best-described lysosomal cysteine peptidase is the cathepsin L-like protease, CPL-1 [15], [16]. We designed a yellow-fluorescent protein (YFP) tagged version of full-length CPL-1 (CPL-1::YFP), and mutated residues in the prepro-domain. While the wild-type CPL-1::YFP targeted to lysosomal-like structures, the mutant form accumulated in the ER upon inhibition of ERAD or UPS. This sensor for ERAD or UPS inhibition was easily detected using widefield epifluorescence microscopy and basic biochemical methods. Taken together, these studies suggest that transgenic animals expressing the mutated form of CPL-1::YFP will serve as a useful tool for conducting high-throughput genetic or pharmacologic screens for modifiers of the metazoan ERAD and UPS pathways.

## Results

### Mutations in the prepro-domain of *C. elegans* CPL-1 prevents trafficking to the endo-lysosomal compartment

Initially, we sought to identify a *C. elegans* orthologue of yeast CPY. We identified 6 genes with approximately 30% and 48% similarity to yeast CPY and its human homologue, cathepsin A, respectively (Figure S1A). However, none of the genes encoded the 91 amino acid pro-domain of CPY or the 2-kDa internal excision fragment of cathepsin A that are required for proper folding and activation, suggesting that the *C. elegans* proteins may be processed differently [17]. We cloned one of these wild-type genes (F13D12.6) and fused it to the N-terminus of YFP, as has been described for CPY-like or cathepsin A transgenes [18], [19]. However, transgenic animals harboring the wild-type transgene, as well as those with a mutation corresponding to that in CPY* (G166R in F13D12.6), yielded a diffuse reticular pattern consistent with localization to the ER, but not the expected localization to lysosomal structures or dilated ER, respectively (Figure S1B–I). This finding suggested either F13D12.6 does not have the same subcellular distribution as CPY and cathepsin A, or the transgene yielded an aberrant protein that was not targeted to their proper locations. DNA sequencing of the transgenes did not reveal any abnormalities within the F13D12.6 genomic regions (not shown) and an immunoblot revealed a fusion protein of the correct molecular mass (Figure S1J). There was also no increase in fluorescence of the mutant protein upon ERAD inhibition, as would be expected if it were an ERAD substrate (Figure S1K).

**Figure 1:**
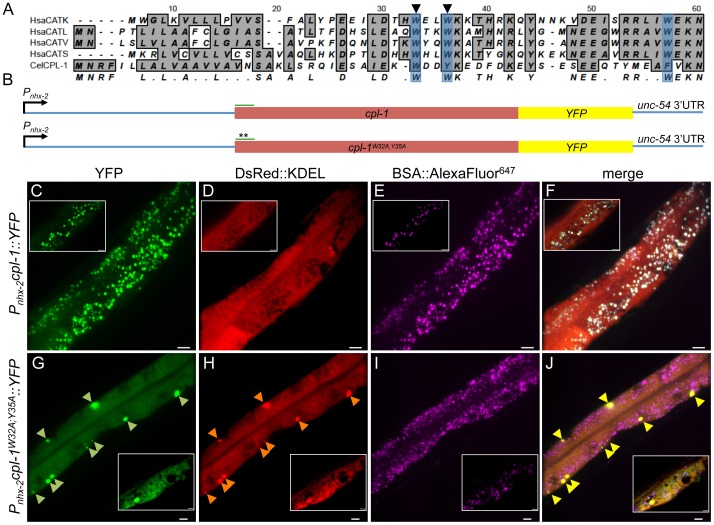
Mutations in the prepro-domain of CPL (CPL-1^W32A;Y35A^) cause ER accumulation and prevent trafficking to the lysosome. (A) Alignment of the primary amino acid sequence from *C. elegans* (Cel) CPL-1[NP_507199.1] with human (Hsa) cathepsins K [AAH16058.1] (CATK), L [NP_666023.1] (CATL), S [AAC37592.1] (CATS) and V [BAA25909.1] (CATV) using the ClustalW algorithm. Blue shading indicates the three tryptophan residues within the human CATL-like prepro-domain that are critical for proper folding [14]. Arrowheads indicate the residues mutated to alanines in the CPL-1 sequence to generate CPL-1^W32A;Y35A^::YFP. [ ] denote accession numbers of individual amino acid sequences used in alignments. (B) Schematic representation of the expression constructs used to express either wild-type or mutant CPL-1::YFP. The asterisks denote location of the mutated resides within the prepro-domain (green line). The intron locations were not depicted. (C–J) Transgenic animals expressing CPL-1::YFP (C–F) or CPL-1^W32A;Y35A^::YFP (G–J) were examined by confocal microscopy and maximum intensity projections are displayed. Both lines were also co-injected with a DsRed::KDEL transgene to mark the ER (D, H), and were also incubated with BSA::AlexaFluor^647^ to label the endo-lysosomal compartment (E, I). CPL-1::YFP showed a punctate distribution within intestinal cells (C) that co-localized with BSA::AlexaFluor^647^ (E, F), but did not overlap with DsRed::KDEL (D). This pattern suggested CPL-1::YFP was trafficking correctly to the endolysosomal compartment. In contrast, CPL-1^W32A;Y35A^::YFP displayed a fine reticular pattern (G, inset) with a few intracellular inclusions (G, arrowheads) that co-localized with the DsRed::KDEL ER marker (H and J), but not the BSA::AlexaFluor^647^ endo-lysosomal marker (I). Insets of single z plane images are included to highlight the distinct reticular fluorescence pattern displayed by the DsRed::KDEL ER marker and the YFP fluorescence pattern observed in animals expressing CPL-1^W32A;Y35A^::YFP. Scale bar represents 10 µm.

Rather than determine whether the expression pattern for wild-type and mutant F13D12.6 was accurate or artifactual, we turned our attention to the highly homologous papain-like cysteine peptidase family. Mutations in any one of three conserved tryptophan residues in the prepro-domain of cathepsin L-like lysosomal cysteine peptidases destabilizes the alpha-helical motif resulting in misfolding and elimination from the ER via ERAD and the UPS [14]. To determine if the *C. elegans* cathepsin L-like protease, CPL-1, could be mutated in a similar fashion, we aligned the first 60 amino acids of the pro-domain of *cpl-1* with those from the human cathepsin L-like cysteine proteases, cathepsins K, L, S and V (Figure 1A). This alignment revealed the presence of conserved tryptophan or bulky hydrophobic residues in the region essential for the formation of the hydrophobic stack that facilitates proper folding of the protease (Figure 1A, blue shading) [14]. For simplicity, we generated a prepro-domain double mutant (W35A and Y35A) of *cpl-1* (Figure 1A, arrowheads), and inserted the entire wild-type or mutated gene between the promoter of *nhx-2* and YFP to yield vectors containing P*_nhx-2_cpl-1::YFP* and P*_nhx-2_cpl-1^W32A;Y35A^::YFP*, respectively (Figure 1B). We chose the intestinal-cell specific *nhx-2* promoter [20], [21], since intestinal expression is easy to visualize under low power microscopy and intestinal cells are a rich biosynthetic source of lysosomal cysteine peptidases [10], [11].

To determine the subcellular localization of CPL-1 by confocal microscopy, we generated transgenic lines by injecting either the wild-type or mutant form of the CPL-1 constructs along with the ER-localization marker, P*_nhx-2_DsRed::KDEL*, and fed them on plates containing the fluid-phase endolysosomal marker, BSA::AlexaFluor^647^. Wild-type CPL-1::YFP appeared as discrete puncta within intestinal cells (Figure 1C) and co-localized with the endo-lysosomal marker BSA::AlexaFluor^647^, but not with the ER-retained DsRed::KDEL (Figure 1C–F). In contrast, CPL-1^W32A;Y35A^::YFP was distributed in a more reticular pattern (Figure 1G, inset) with accumulations (Figure 1G, arrowheads) that co-localized with the ER marker, but not the endo-lysosomal marker (Figure 1H–J). These data were consistent with that from a mammalian cell culture system demonstrating that a combination of the W28A and W31A mutations causes pro-cathepsin S misfolding, retention within the ER, and loss of endo-lysosomal targeting [14].

Conceivably, the differences in the subcellular localization between the wild-type and mutant forms of CPL-1 could be secondary to differential effects of the transgenes on overall health and viability of the animals or marked variation in transgene expression. However, the longevity of the transgenic animals did not differ from that of the wild-type N2 animals (Figure S2), nor was there any visual evidence of morphological abnormalities as assessed by DIC microscopy (not shown).

**Figure 2:**
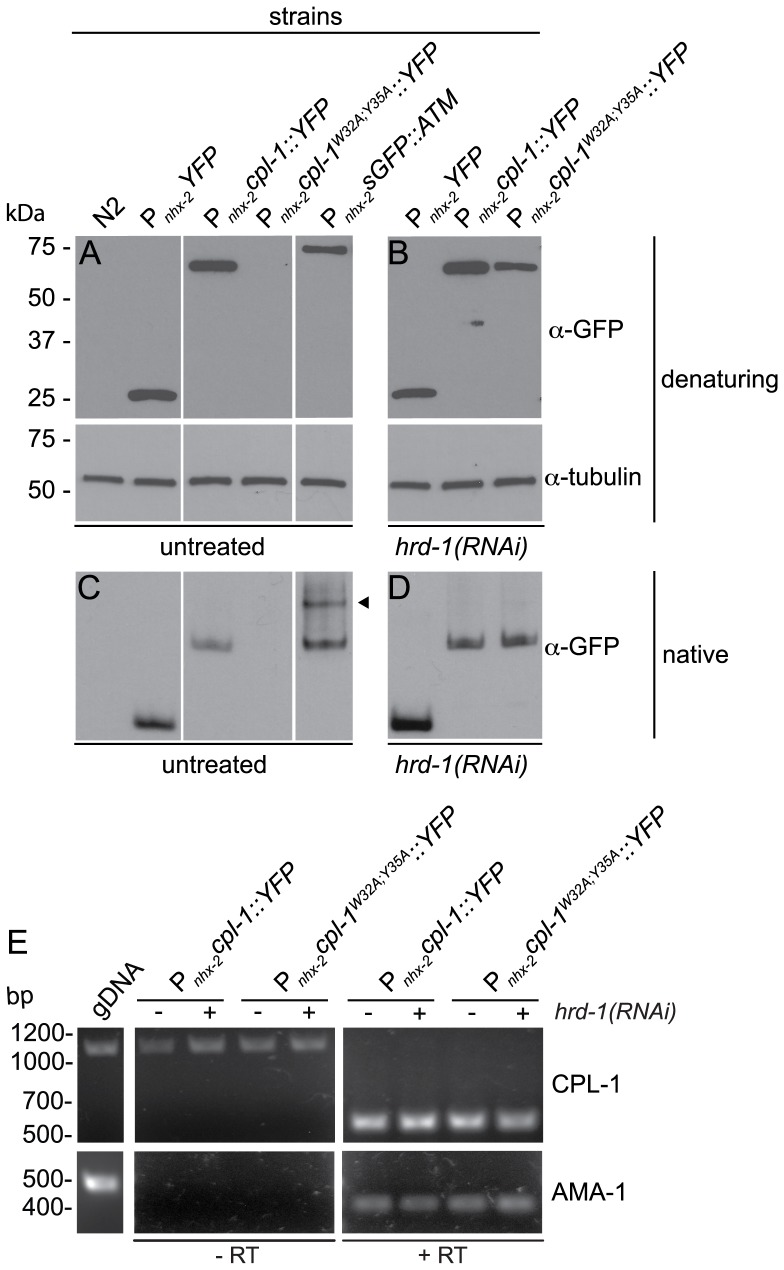
CPL-1 and CPL-1^W32A;Y35A^ protein and mRNA expression. (A–D) Immunoblots of total protein lysates derived from wild-type (N2) or transgenic strains that were unexposed (A, C) or exposed to *hrd-1(RNAi)* (B, D). Protein lysates, separated by either SDS- (A–B) or native PAGE (C–D), were immunoblotted with anti-GFP polyclonal antisera that detects both YFP and GFP. Furthermore, the membranes from the SDS-PAGE were stripped and re-probed with α-tubulin monoclonal antibody to control for protein loading. Unlike CPL-1::YFP, was CPL-1^W32A;Y35A^::YFP was detected under denaturing (and native gel) conditions only after ERAD inhibition by *hrd-1(RNAi)*. As compared to the polymerizing GFP::ATM control (arrowhead), neither CPL-1 protein appeared to form higher order polymers as detected by native PAGE. (E) Steady-state CPL-1 mRNA (514 bp) levels. Total RNA isolated from 350 P*_nhx-2_cpl-1::YFP;*P*_myo-2_mCherry* or P*_nhx-2_cpl-1^W32AY35A^::YFP;*P*_myo-2_mCherry transgenic* animals, treated with either *vector* or *hrd-1(RNAi)*, was assessed by reverse transcriptase (RT) PCR (RT-PCR). No RT, genomic DNA (gDNA) template and primers for a housekeeping cDNA, AMA-1, (425 bp) served as controls. Diluted CPL-1 mRNA levels derived from the different transgenic strains were comparable.

To determine if the effects of the W32A and Y35A mutations on CPL-1 were due to quantitative or qualitative (e.g., a truncated or polymerizing protein) changes in protein expression, whole animal lysates were analyzed under denaturing and non-denaturing conditions using PAGE and immunoblotting with anti-GFP/YFP antisera (Figure 2). Under denaturing conditions, the appropriate size bands were detected in lysates from P*_nhx-2_YFP* (∼28-kDa), P*_nhx-2_cpl-1::YFP* (∼75-kDa), and a control line expressing human α1-antitrypsin, P*_nhx-2_sGFP::ATM* (∼75-kDa) (Figure 2A) [21]. However, no protein was detected in the lysates in from the P*_nhx-2_cpl-1^W32A;Y35A^::YFP* line. This result what not surprising, as the mutant protein might be rapidly degraded, and below the limit of detection by immunoblotting. Alternatively, the *cpl-1* containing transgenes might be differentially expressed. To test this hypothesis, we performed semi-quantitative, RT-PCR on RNA samples from the transgenic lines (Figure 2E). There appeared to be no appreciable difference in steady state mRNA levels (Figure 2E), suggesting that CPL-1^W32A;Y35A^::YFP was being rapidly degraded. If rapid degradation was the cause, then inhibition of the CPL-1^W32A;Y35A^::YFP elimination pathway should lead to increased CPL-1^W32A;Y35A^::YFP accumulation. This appeared to be the case (Figure 2B) and will be described further in the next section.

Transgenic line lysates were also subjected to native PAGE to determine whether the difference between wild-type or mutant CPL-1 expressing lines resulted from excessive polymer formation. In comparison to the sGFP::ATM controls, which formed both monomeric and dimeric species (Figure 2C, arrowhead) [22], neither CPL-1 protein produced polymers under these lysis conditions, although the CPL-1^W32A;Y35A^::YFP band was only visible after ERAD inhibition (*vide infra*) (Figure 2C–D). These studies suggested that both the wild-type and mutant *cpl-1* containing transgenes yielded full-length proteins, at comparable levels, and that their expression had no adverse effects on the development or survival of the transgenic lines.

### CPL-1^W32A;Y35A^ accumulated in the ER upon ERAD inhibition

**Figure 3:**
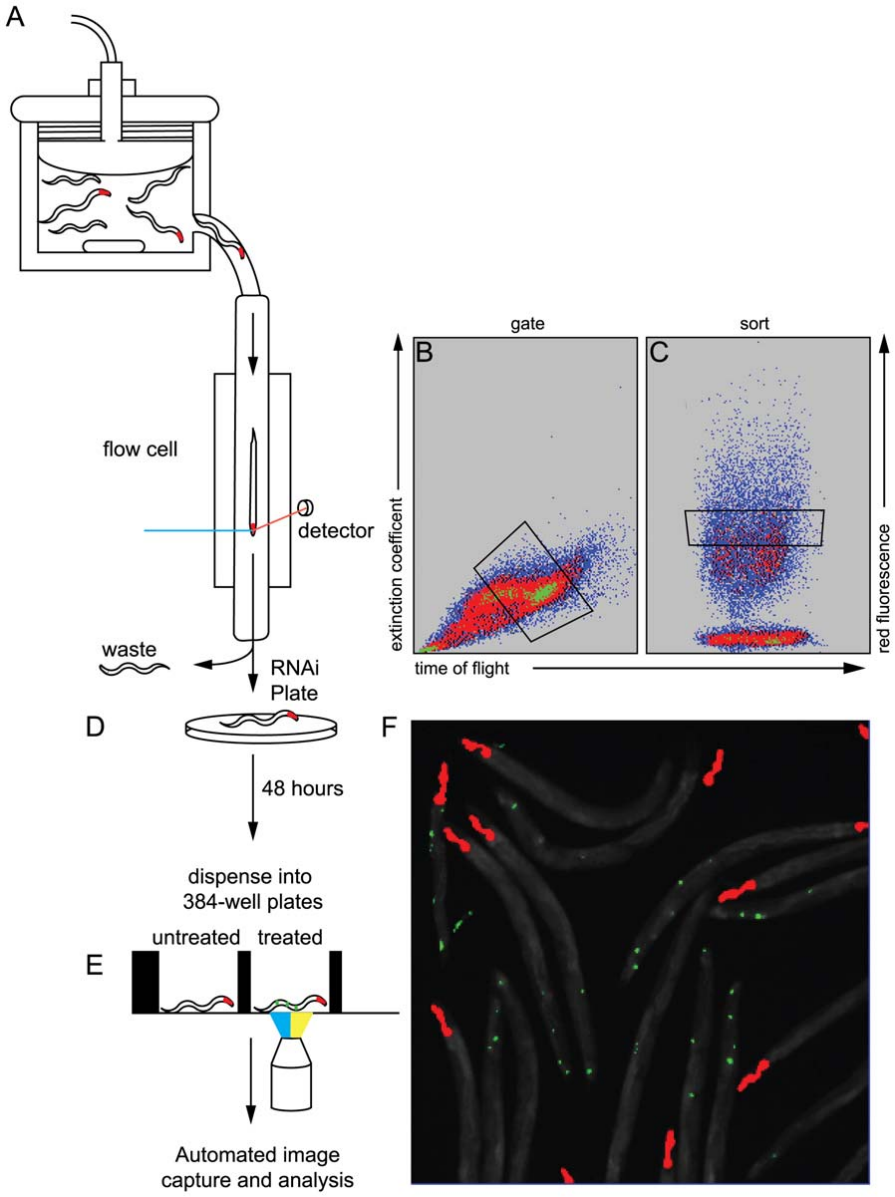
Workflow used to identify changes in CPL-1^W32A;Y35A^::YFP accumulation after exposure to different RNAi treatments. (A–C) Synchronized animals were collected in the COPAS Biosort sample cup (A) and passed through a flow cell, where L4 staged animals were gated by a combination of extinction coefficient and time of flight (TOF) (B). A subset of the gated L4 animals was selected on the basis of red fluorescence and TOF (sorted region) through the flow cell (C). (D) Selected animals were dispensed onto NGM plates seeded with *E. coli* expressing double stranded RNAs. (E) After 48 hours, animals were collected and dispensed into a 384-well optical bottom plate for fluorescence quantification using the ArrayScan V^Ti^ automated microscope and analysis system. (F) The number of animals in each well were counted by using the mCherry head marker (red) to identify individual animals while the GFP channel was used to identify the number, intensity and size of the CPL-1^W32A;Y35A^::YFP accumulations (green). The total area of CPL-1^W32A;Y35A^::YFP accumulations per worm was calculated by dividing the total area of YFP fluorescence by the total number of mCherry heads identified in each well. Fold-increases values were determined by normalizing to the vector RNAi in order to account for day-to-day variations in transgene expression levels. The experiments were performed in triplicate and displayed as an average of the three trials ± the standard error of the mean (SEM).

Inhibition of the ERAD machinery should lead to further accumulation of CPL-1^W32A;Y35A^::YFP if it was a luminal substrate. To test this hypothesis, we subjected the transgenic lines and their controls to *ERAD(RNAi)* by feeding a subset of bacterial clones from the Ahringer library that express double-stranded RNAs to different ERAD components (Table 1) [23]. To decrease inter-assay variability in protein expression associated with non-integrated transgenes, and to make the assessments of the RNAi effects more quantitative, we employed the COPAS BioSort large particle flow cytometer to help set-up the assays and the ArrayScan^VTi^ to automate image acquisition and data analysis, respectively (Figure 3) [21]. Also, we generated a second set of transgenic animals by co-injecting either P*_nhx-2_cpl-1::YFP* or P*_nhx-2_cpl-1^W32A;Y35A^::YFP* with the pharyngeal marker, P*_myo-2_mCherry*. As previously described [21], this latter transgene drives mCherry expression in the pharynx and is used as an internal standard for the possible changes in CPL-1^W32A;Y35A^::YFP expression due to nonspecific RNAi effects. In addition, selective mCherry expression in the pharynx facilitates the selection of stage-specific transgenic animals and autofocusing using the BIOSORT and ArrayScan^VTi^, respectively [21].

**Table 1:** *C. elegans* ERAD and UPS genes selected for RNAi.

*C. elegans* Gene	Human Orthologue	Function	References
**ERAD**			
*cdc-48.1* and *48.2*	p97/VCP	AAA+ ATPase, retro-translocation of misfolded proteins	[24], [48], [49]
*npl-4.1* and *4.2*	NPL4	binding partner with CDC-48.1 and .2	[24]
*ufd-1*	UFD1	binding partner with CDC-48.1 and .2	[24], [49]
*ufd-2*	ubiquitination factor E4B	E4 ubiquitin conjugation factor	[13], [50], [51]
*cup-2*	Derlin1	receptor for VCP/p97/cdc-48	[52], [53]
*sel-11/hrd-1*	HRD1/synoviolin	ER-membrane resident ubiquitin ligase	[13], [50]
*sel-1*	SEL1	member of the HMG-CoA reductase Degradation (HRD) complex	[24], [53]
*hrdl-1*	AMFR/gp78	ER-membrane resident ubiquitin ligase	[13], [50]
**UPS**			
*rpn-2*	PSMD1/Rpn2	non-ATPase subunit of the 26S proteasome's 19S regulatory particle	[54], [55]
*rpn-10*	Rpn10A	non-ATPase subunit of the 26S proteasome	[13], [54], [55]
*rpt-5*	PSMC3/TBP1	AAA ATPase subunit of the 26S proteasome's 19S regulatory particle	[13], [54], [55]
*pas-4*	PSMA2/HC3	proteasome alpha subunit	[55]
*pbs-2*	PSMB7	proteasome beta subunit	[55]

**Figure 4:**
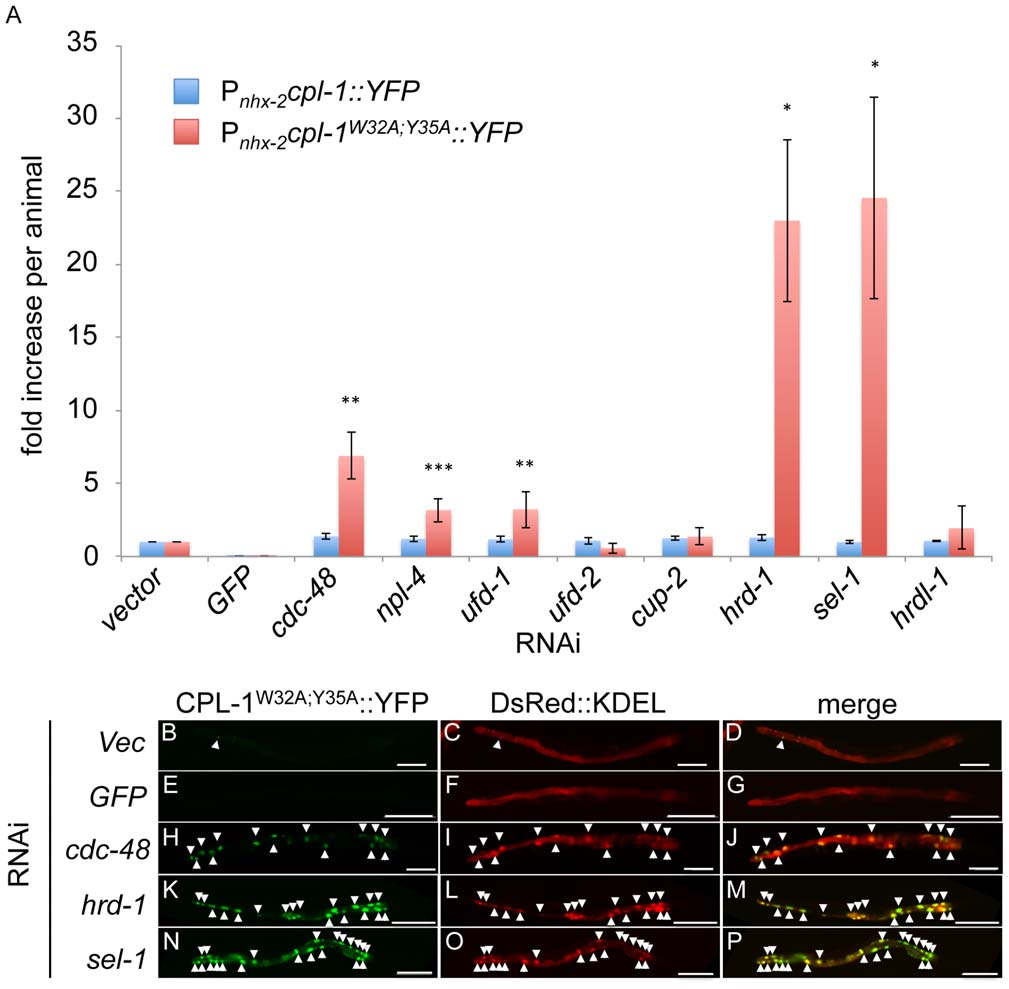
CPL-1^W32A;Y35A^::YFP accumulated after knockdown of ERAD components. (A) Either P*_nhx-2_cpl-1::YFP;*P*_myo-2_mCherry* or P*_nhx-2_cpl-1^W32AY35A^::YFP;*P*_myo-2_mCherry* animals were treated with RNAi and analyzed as described in Figure 3. Statistical analysis of the RNAi treated animals relative to vector was performed using an unpaired, 2-tailed *t*-test (unequal variance) (*p<0.05, **p<0.01, ***p<0.001). (B–P) P*_nhx-2_cpl-1^W32AY35A^::YFP;*P*_myo-2_mCherry;DsRed::KDEL* animals were exposed to *vector (B–D), GFP (E–G)*, *cdc-48 (H–J)*, *hrd-1 (K–M)* or *sel-1 (N–P)* RNAi for 48 h. and images were collected using a widefield epifluorescence microscope. The arrowheads indicate accumulations of CPL-1^W32A;Y35A^, which co-localized with the ER marker, DsRed::KDEL. Scale bar indicates 100 µm.

Transgenic animals expressing CPL-1^W32A;Y35A^::YFP treated with RNAi's directed against *cdc-48*, *npl-4*, *ufd-1*, *hrd-1* or *sel-1* showed significant accumulation of the mutant protein as measured quantitatively by the ArrayScan^VTi^ (Figure 4A). The same RNAi's had no effect on the steady-state levels of CPL-1::YFP (Figure 4A) or YFP (Figure S3A), suggesting that the RNAi effects on CPL-1^W32A;Y35A^::YFP expression were not due to an unanticipated or indirect effects that generally enhanced CPL-1 mRNA stability or increased *nhx-2* promoter activity, respectively. To control for the *ERAD(RNAi)* that appeared to have no effect on CPL-1^W32A;Y35A^::YFP accumulation, we tested their ability to activate the unfolded protein response (UPR) using the *ire-1* activation sensor, P*_hsp-4_GFP* (Figure S3B). Consistent with published data, all of the ERAD RNAi's tested, except for *hrdl-1(RNAi)*, significantly increased GFP expression in transgenic animals carrying the P*_hsp-4_GFP* transgene (Figure S3B) [24]. To confirm that *hrdl-1(RNAi)* was active, we performed semi-quantitative reverse transcriptase PCR and showed that steady-state HRDL-1 mRNA levels were decreased in P*_nhx-2_cpl-1^W32A;Y35A^::YFP* animals treated with *hrdl-1(RNAi)* but not *control(RNAi)* (Figure S3C).

Since CPL-1^W32A;Y35A^::YFP fluorescence increased with ERAD inhibition, we determined whether ERAD activity accounted for the inability to detect CPL-1^W32A;Y35A^::YFP by immunoblotting. Protein lysates from CPL-1^W32A;Y35A^::YFP transgenic animals treated with *hrd-1(RNAi)* had detectable proteins levels, under both denaturing and non-denaturing conditions, comparable to those of the CPL-1::YFP expressing line (Figure 2B, D). These data suggested that ERAD was responsible for decreasing the steady-state levels of CPL-1^W32A;Y35A^::YFP.

To confirm that ERAD inhibition resulted in CPL-1^W32A;Y35A^::YFP accumulation within the ER (Figure 4B–P), we repeated the studies using transgenic animals co-expressing DsRed::KDEL. Single plane widefield epifluorescence images of the entire animal (n = 5–10) were obtained using constant image acquisition settings. Animals treated with *vector(RNAi)*, as a negative control, showed little accumulation of CPL-1^W32A;Y35A^::YFP (Figure 4B) that co-localized with the DsRed::KDEL ER marker (Figure 4C–D, arrowhead). *GFP(RNAi)*, reduced the YFP signal such that it is undetectable under these imaging conditions (Figure 4E–G). In contrast, there was a substantial increase in both the intensity and the number of YFP accumulations (arrowheads) that co-localized with the DsRed::KDEL ER marker when these animals were exposed to *cdc-48(RNAi)* (Figure 4H–J), *hrd-1(RNAi)* (Figure 4K–M) or *sel-1(RNAi)* (Figure 4N–P). Taken together, these data suggested that CPL-1^W32A;Y35A^ accumulated within the ER upon ERAD inhibition. Furthermore, these data provided direct evidence that components of the mammalian ERAD pathway, involved in the degradation of ER luminal substrates, were conserved in *C. elegans*.

### Proteasomal inhibition enhanced CPL-1^W32A;Y35A^ accumulation

Since proteasomal degradation is the end-point of the ERAD pathway, its inhibition should result in CPL-1^W32A;Y35A^::YFP accumulation. To test this hypothesis, we examined animals after proteasomal inhibition by both RNAi's directed at different proteasomal subunits (Table 1) and chemical inhibitors of proteasomal catalytic activity. As a positive control for proteasomal activity, we generated a transgenic line carrying the ubiquitin (UB)-fusion-degradation (UFD) transgene, P*_nhx2_UB-V::mCherry*. The UB-V::mCherry fusion protein contains a G76V mutation which blocks de-ubiquitination by de-ubquitinating enzymes (DUBs) and results in constitutive degradation by the proteasome [25]. As a negative control, we generated a transgenic line that expresses UB-M::mCherry (containing a R77M mutation), which allows for the removal of the ubiquitin moiety by DUBs but prevents subsequent re-ubiquitination. This mutation prevents proteasomal degradation and results in constitutive cytoplasmic expression regardless of proteasomal activity [25]. To ensure proper transgenic selection, image acquisition and analysis; these two ubiquitin expression constructs were co-injected separately with P*_myo-2_GFP*.

**Figure 5:**
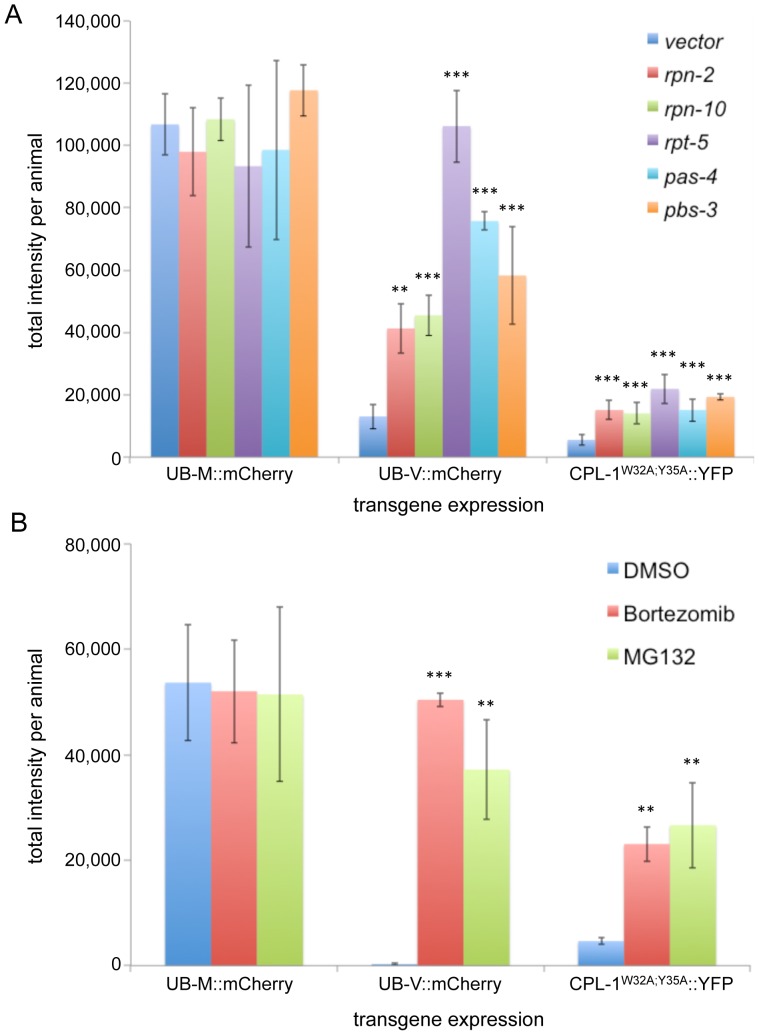
CPL-1^W32A;Y35A^::YFP accumulation after proteasomal inhibition. UB-M::mCherry, UB-V::mCherry, or CPL-1^W32A;Y35A^::YFP expressing transgenic animals were exposed to either a proteasomal RNAi panel (A) or chemical inhibitors (B). Animals were processed as described in Figure 3. For the UB-M::mCherry and UB-V::mCherry expressing animals, the algorithm was adjusted to detect the entire intestinal fluorescence pattern above that of the *vector(RNAi)* control. Total intensity was used in place of total area. Statistical analysis of the RNAi treated animals relative to vector was performed using an unpaired, 2-tailed *t*-test (unequal variance) (**p<0.01, ***p<0.001). Both proteasomal RNAi and chemical inhibitors caused a significant increase in in CPL-1^W32A;Y35A^::YFP fluorescence.

The UFD controls and CPL-1^W32A;Y35A^::YFP transgenic lines were exposed to RNAi's specific for different components of the 19S regulatory particle and the 20S catalytic core for 24 hours (Figure 5A). Since UB-M::mCherry and UB-V::mCherry, unlike the CPL-1^W32A;Y35A^::YFP, were cytosolic proteins with a diffuse distribution, the analysis algorithms were adjusted to distinguish the entire intestine above the background threshold so that the total fluorescence per animal could be determined. Transgenic animals expressing UB-M::mCherry exposed to *vector(RNAi)* had approximately 10-fold higher levels of total intestinal fluorescence within the intestine compared to animals expressing the UB-V::mCherry under the same conditions (Figure 5A). Treatment of the transgenic animals expressing UB-V::mCherry with any one of the proteasomal RNAi's increased the total mCherry fluorescence significantly, but as expected, had no effect on the UB-M::mCherry expressing animals (Figure 5A). This result suggested that knockdown of the selected proteasomal subunits of the regulatory particle or catalytic core reduced the activity of the proteasome. Similarly, CPL-1^W32A;Y35A^::YFP fluorescence was significantly increased when treated with the same proteasomal RNAi panel as compared to *vector(RNAi)* controls (Figure 5A).

While RNAi knockdown prevents new proteasomal subunits from being synthesized, it does not prevent degradation of substrates by preexisting, active proteasomal complexes. To inhibit all proteasomal activity, we utilized the proteasomal inhibitors bortezomib and MG132. Bortezomib has higher specificity for the proteasome than MG132, which also inhibits calpains and lysosomal cysteine proteases [26]–[28]. In both the UB-V::mCherry and CPL-1^W32A;Y35A^::YFP expressing transgenic lines, treatment with either inhibitor increased the levels of total fluorescence significantly when compared to the DMSO control (Figure 5B). As expected, neither of the compounds had an effect on the steady-state levels of the negative control, UB-M::mCherry (Figure 5B). Taken together, these data suggested CPL-1^W32A;Y35A^::YFP accumulated within the animals upon proteasomal inhibition.

### Autophagy inhibition did not increase CPL-1^W32A;Y35A^ accumulation

**Figure 6:**
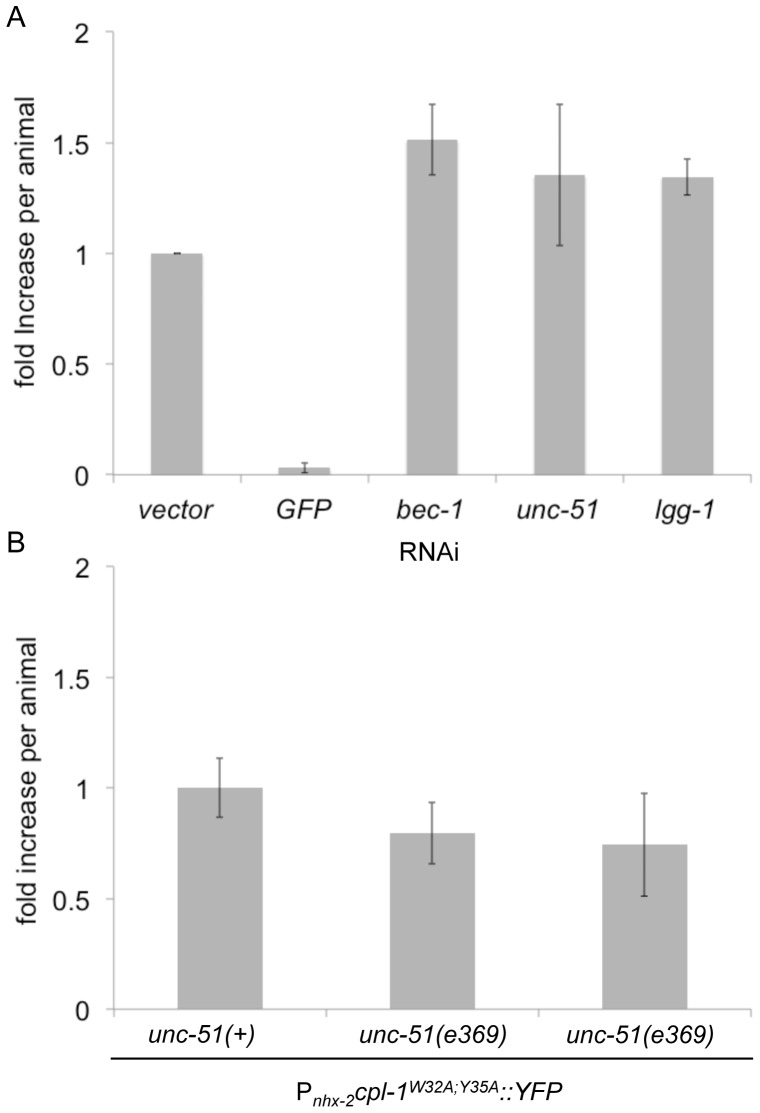
Autophagy inhibition did not affect steady-state levels of CPL-1^W32A;Y35A^::YFP. (A) CPL-1^W32A;Y35A^::YFP animals were exposed to an autophagy RNAi panel and analyzed as described in Figure 3. Statistical analysis of the RNAi treated animals relative to vector was performed using an unpaired, 2 tailed t-test (unequal variance). No significant difference was observed. (B) VK1879 (N2;*vkEx1879[*P*_nhx-2_cpl-1^W32AY35A^::YFP;*P*_myo-2_mCherry]*) animals were crossed with the autophagy-deficient knockout strain *unc-51(e369)* to derive *unc-51(e369);vkEx1879[*P*_nhx-2_cpl-1^W32AY35A^::YFP;*P*_myo-2_mCherry]*. Two individual lines (VK1984 and VK1985) were selected and analyzed as described in Figure 3. Results were compared to those obtained in the original CPL-1^W32A;Y35A^::YFP strain. No significant difference in CPL-1^W32A;Y35A^::YFP expression was observed in the autophagy deficient strains.

Although our data suggested that CPL-1^W32A;Y35A^::YFP was a luminal ERAD substrate, some misfolded proteins are eliminated by both the autophagic and ERAD pathways [29], [30]. To determine whether autophagy might also play a role in the elimination of this mutant protein, we exposed CPL-1^W32A;Y35A^::YFP expressing animals to RNAi's specific for several different genes required for autophagy; *bec-1*, *unc-51*, and *lgg-1* [31]. Knockdown of these genes did not significantly increase the accumulation of CPL-1^W32A;Y35A^::YFP, whereas *GFP(RNAi)* reduced the signal to nearly undetectable levels (Figure 6A). To confirm that the autophagy pathway was inhibited upon exposure to these RNAi's, transgenic animals expressing mCherry::LGG-1, under control of the *nhx-2* promoter, were treated with identical RNAi's and then starved for 4 hours as previously described ([21] and Figure S4). Starvation is a potent inducer of autophagosome formation and results in a change in LGG-1 distribution from diffuse to punctate, as this protein becomes incorporated into autophagosomal structures. As shown in the vector control, mCherry::LGG-1 was visualized as discrete puncta in the posterior intestine after starvation (Figure S4, inset); whereas mCherry::LGG-1 displayed a more diffuse cytoplasmic expression pattern in the *bec-1(RNAi)* and *unc-51(RNAi)* treated worms. These results suggested that these RNAi treatments blocked autophagosome formation. Additionally, treatment with *lgg-1(RNAi)* suppressed the expression of mCherry::LGG-1, showing that LGG-1 expression itself could be down-regulated. Although there was no significant increase in CPL-1^W32A;Y35A^::YFP accumulation after autophagy knockdown, there was a slight increase in YFP fluorescence compared to control animals. To confirm that the autophagy pathway was not a major means of CPL-1^W32A;Y35A^::YFP disposal, we crossed P*_nhx-2_cpl-1^W32A;Y35A^::YFP* animals with an *unc-51(e369)* knockout strain to yield *unc-51(e369*);P*_nhx-2_CPL-1^W32AY35A^::YFP;*P*_myo-2_mCherry* animals. Two independent lines were analyzed, and showed no significant differences in the level of CPL-1^W32A;Y35A^::YFP, as compared to the controls (Figure 6B). These data suggested that inhibition of the autophagy pathway had no detectable effect on the disposal of CPL-1^W32A;Y35A^::YFP.

## Discussion

*C. elegans* has become one of the preferred model systems to study cell biological processes due to its genetic tractability and adaptability to high throughput screening platforms [32]. However, its usefulness as a model for studying ERAD has been hindered by the lack of well-characterized luminal substrates that permit the process to be tracked biochemically, or microscopically in real-time. Misfolded secretory proteins have been shown to be ERAD substrates, including papain-like lysosomal cysteine peptidases with prepro-domain mutations [33], [34]. Mutations of the conserved tryptophan residues in the prepro-domain of cathepsin S results in misfolding and degradation via the ERAD pathway with a half-life similar to the canonical ERAD substrate, yeast CPY* [14], [35]. Therefore, we determined whether mutated CPL-1 was a luminal ERAD substrate in *C. elegans*. CPL-1 was selected for three reasons. First, CPL-1 has high homology to the human cathepsins K, L, S and V. Second, this protein is ubiquitously expressed (including intestine) and is known to function in embryogenesis, yolk protein processing, molting and lysosomal-dependent necrotic cell death pathway [15], [16], [36]–[39]. Third, the prepro-domain of CPL-1 contains bulky hydrophobic amino acids (W32 and Y35) at positions identical to those mutated in cathepsin S [14], suggesting that the conformation of the prepro-domain of CPL-1 has folding properties similar to those of human cathepsins. Our studies show that wild-type CPL-1::YFP was targeted to the endo-lysosomal compartment in intestinal cells. Interestingly, and as is common for many cathepsins, a significant fraction of the CPL-1::YFP was also secreted, as evidenced by YFP fluorescence in the pseudocoelomic space (data not shown), where it was taken up by oocytes, and subsequently appeared in the eggs (the *nhx-2* promoter does not drive embryonic expression) [20], [21]. These results suggested that CPL-1::YFP was transported correctly and the YFP tag does not perturb delivery to the endo-lysosomal compartment and the pseudocoelomic space. In contrast, the W32A;Y35A mutations in the prepro-domain of the CPL-1 (CPL-1^W32A;Y35A^) abolished the ability of CPL-1 to traffic to these locations. Furthermore, the YFP signal was visible in the ER, either as a diffuse reticular pattern or within distended cisternae (Figure 1). This result suggested that the CPL-1^W32A;Y35A^::YFP was misfolded and was no longer capable of traversing the classical secretory pathway. As a consequence of its ER retention, we hypothesized that CPL-1^W32A;Y35A^::YFP was being degraded by ERAD. This notion was supported by RNAi experiments showing that ERAD inhibition enhanced the accumulation CPL-1^W32A;Y35A^::YFP within the ER. Not surprisingly, inhibition of different ERAD components increased the accumulation CPL-1^W32A;Y35A^::YFP to a different extent. These variations reflect the selective importance of different ERAD components in handling various substrates, as well as technical features associated with differences in RNAi efficacy or differential protein half-lives of ERAD components. Nonetheless, inhibition of the E3 ligase, HRD-1 and one of its binding partners, SEL-1, were the highest inducers of CPL-1^W32A;Y35A^::YFP accumulation, and based on their homology to their human and yeast counterparts, are likely to be involved in transporting misfolded luminal proteins such as CPL-1^W32A;Y35A^::YFP to the cytosol [40]. Also CDC-48, and its binding partners, NPL-4 and UFD-1 led to a significant increase in CPL-1^W32A;Y35A^::YFP accumulation. CDC-48 and its cofactors are cytosolic proteins that extract ubiquitinated ERAD substrates from the ER membrane complexes and delivery their cargo to the proteasome for degradation [41].

**Figure 7:**
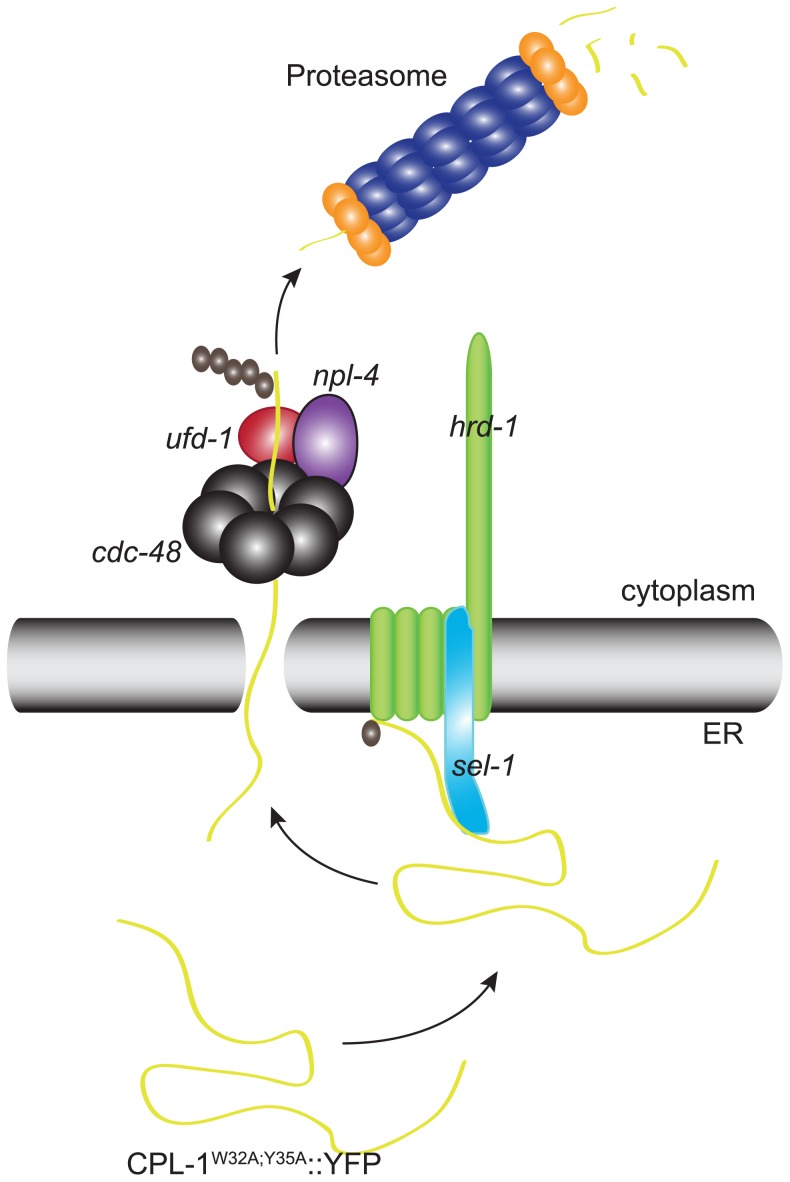
Schematic representation of CPL-1^W32A;Y35A^::YFP degradation by ERAD. Misfolded CPL-1^W32A;Y35A^::YFP is recognized and targeted to the *hrd-1*/*sel-1* complex where it is ubiquitinated. Following polyubiquitination CPL-1^W32A;Y35A^::YFP is retro-translocated by the CDC-48^NPL-4/UFD-1^ complex and degraded by the proteasome.

Since the proteasome is the final destination for most soluble ERAD substrates, we present data consistent with CPL-1^W32A;Y35A^::YFP being degraded by the proteasome and not by autophagy. Inhibition of proteasomal function by RNAi directed against specific proteasomal subunits or chemical inhibitors also resulted in a marked accumulation of CPL-1^W32A;Y35A^::YFP. Taken together, these findings suggested that CPL-1^W32A;Y35A^::YFP was a luminal ERAD substrate (Figure 7).

The canonical ERAD substrate is the mutated yeast vacuolar protease, CPY* [10]. There are at least six *C. elegans* proteins that have varying degrees of homology to yeast CPY based on BLAST algorithms (WormBase web site, http://www.wormbase.org, release WS228, 01/20/12), with the most homologous, F13D12.6, showing the greatest similarity (33.4%) and identity (19.7%). However, F13D12.6::YFP did not traffic to the endo-lysosomal compartment. Since *C. elegans* intestinal cells contain both lysosomes and lysosomal-related organelles [42], it is conceivable that F13D12.6 was targeted to a subset of organelles that do not originate or merge with the endo-lysosomal compartment. Moreover, a G166R mutation within the putative active site of F13D12.6 (equivalent to the active site mutation of yeast CPY*) did not cause accumulation within the ER after ERAD inhibition. These findings suggested that mutant F13D12.6, as constructed by our laboratory, was not eliminated by ERAD.

In summary, upon ERAD/UPS inhibition, CPL-1^W32A;Y35A^::YFP accumulation was easily discernible by widefield epifluorescence microscopy. Thus, the animals expressing CPL-1^W32A;Y35A^::YFP should facilitate the use of high-throughput chemical or genome-wide genetic screens to further our understanding of the ERAD and UPS pathways.

## Materials and Methods

### Construction of expression plasmids

All amplifications were performed using the Expand high fidelity PCR system (Roche Applied Science, Indianapolis, IN). Restriction enzymes used for cloning procedures were purchased from New England Biolabs (NEB, Ipswich, MA) unless otherwise stated.

To generate the P*_myo-2_* mCherry construct (pAV1944) (Table S1), a 717 bp mCherry cDNA was PCR amplified (primer set 1, Table S2), and the fragment sub-cloned into the NheI and EcoRV restriction sites of the canonical expression vector, pPD49.26 [43], to create mCherry_pPD49.26 (pAV1997). A 1.1 kb *myo-2* promoter was amplified (primer set 2), and the fragment was ligated into the SphI and XbaI sites to yield the final construct, pAV1944.

For YFP fusion proteins, an NheI/SacI double digest was performed on P*_nhx-2_mCherry* (pAV1951) followed by gel purification to remove mCherry. Next, an 868 bp YFP cDNA containing synthetic introns was amplified (primer set 3) from pPD133.86 (a kind gift from Dr. Andrew Fire), and the fragment ligated into the NheI and SacI restriction sites to form P*_nhx-2_YFP* (pKS2236). P*_nhx-2_cpl-1::YFP* (pKS2301) was generated by amplifying a 2.6 kb *cpl-1* genomic DNA fragment (primer set 4), and ligating the fragment into the NheI site of P*_nhx-2_YFP* (pKS2236). P*_nhx-2_cpl-1^W32A;Y35A^::YFP* (pKS2311) was created by site-directed mutagenesis of those codons corresponding to amino acids 32 and 35 of P*_nhx-2_ cpl-1::YFP* using the Quikchange™ mutagenesis kit (primer set 5) according to manufacturers instructions (Agilent Technologies, Santa Clara, CA).

P*_nhx-2_F13D12.6::YFP* (pNG2462) was generated by amplifying a 3.2 kb *F13D12.6* genomic DNA fragment (primer set 6) and ligating the fragment into the NheI site of P*_nhx-2_YFP* (pKS2236). P*_nhx-2_F13D12.6^G166R^::YFP* (pNG2470) was generated by site-directed mutagenesis (primer set 7).

To generate P*_nhx-2_DsRed::KDEL* (pAV1825), the *nhx-2* promoter was amplified (primer set 8) and sub-cloned into the HindIII and XbaI sites of pPD95.85. The stop codon of GFP was mutated by Quikchange™ site-directed mutagenesis to a KasI site (primer set 9). Next, the 675 bp DsRed insert was amplified (primer set 10) from the pDsRed-Express-C1 vector (Clontech, Mountain View, CA) with KpnI and KasI sequences at the 5′ and 3′ ends, respectively. The reverse primer also contained the codons corresponding to the ER retention motif, KDEL. The GFP insert was replaced by ligating the DsRed::KDEL insert into the KpnI and KasI sites.

To generate the P*_nhx-2_UB-V::mCherry* (pSG2142) construct, the 248 bp ubiquitin sequence was amplified (primer set 11) from the P*_unc-47_Ub^A47V^::DsRed* expression construct [44] with NheI restriction sites at both 5′ and 3′ ends along with a short linker sequence. The *nhx-2* promoter and amplified ubiquitin sequence were sub-cloned into the HindIII/XbaI and NheI sites, respectively, of mCherry_pPD49.26 (pAV1997). Quikchange™ site directed mutagenesis (primer set 12) of the P*_nhx-2_Ub-V::mCherry* (pSG2142) yielded an intermediate construct, P*_nhx-2_UB-R::mCherry* (pSG2143), which was not used for this investigation. A second round of Quikchange™ site directed mutagenesis (primer set 13) on P*_nhx-2_UB-R::mCherry* (pSG2143) yielded P*_nhx-2_UB-M::mCherry* (pSG2144). The P*_hsp-4_GFP* (pAV2021) construct was generated by amplifying the 1.2 kb *hsp-4* promoter (primer set 14) from genomic DNA and ligating the fragment into the HindIII and XbaI sites of pPD95.77 [43]. The final plasmid constructs (Table S1) were deposited with Addgene (Cambridge, MA).

### *C. elegans* strains and culture conditions

A complete list of worm strains and their genotype, along with the figures they correspond to, is given in Table S3 for easy reference. All injection mixes were made to a final total DNA concentration of 100 ng/µl using pBluescript SK- (Agilent Technologies) if required. The strain VK1104 was generated by co-injecting P*_nhx-2_YFP* and P*_myo-2_mCherry* with 20 ng/µl and 5 ng/µl, respectively, into the gonad of young adult N2 hermaphrodites. Strains VK1256, VK1258, VK1770 and VK1870 were generated by co-injecting 20 ng/µl of the expression plasmids P*_nhx-2_cpl-1::YFP*, P*_nhx-2_ cpl-1^W32A;Y35A^::YFP*, P*_nhx-2_F13D12.6::YFP* and P*_nhx-2_F13D12.6^G166R^::YFP*, respectively, with 20 ng/µl P*_nhx-2_DsRed::KDEL*. Strain VK1879 was generated by co-injecting the P*_nhx-2_ cpl-1^W32A;Y35A^::YFP* plasmid with P*_myo-2_mCherry* at 20 ng/µl and 5 ng/µl, respectively. Strains VK1244, VK1243 and VK1241 were generated by co-injecting 80 ng/µl of the plasmids P*_nhx-2_Ub-M::mCherry*, P*_nhx-2_Ub-V::mCherry* and P*_nhx-2_mCherry::lgg-1*, respectively, with 5 ng/µl of *P_myo-2_GFP*. The worm strain VK737 was generated by co-injecting the P*_hsp-4_GFP* and P*_myo-2_mCherry* plasmids at 80 ng/µl and 5 ng/µl, respectively. Strain VK689 was generated as described by Gosai, et al. [21]. The worm strain VK1984 (*unc-51(e369);vkEx1258[P_nhx-2_CPL-1^W32AY35A^::YFP;P_myo-2_mCherry]*) was generated by crossing males from strain VK1258 (N2; *vkEx1258[P_nhx-2_CPL-1^W32AY35A^::YFP;P_myo-2_mCherry]*) with hermaphrodites from strain CB369 (*unc-51(e369)*), which was obtained from the *Caenorhabditis* Genetics Center (CGC). Males were generated by heat shocking at 27°C for 18 hours. Worms were routinely cultured at 22°C on nematode growth media (NGM) plates seeded with *E. coli* strain OP50 unless otherwise specified. All worm strains generated for this manuscript were deposited at the *Caenorhabditis* Genetics Center (CGC) (Table S3).

### OP50 preparation

OP50 was prepared as described in [45]. Briefly, a single colony of OP50 was placed in 3 ml LB broth and incubated at 37°C with shaking overnight. One ml of this culture was added to 1L of LB broth and was incubated at 37°C until reaching an OD_600_ = 0.5. The bacteria were washed twice with PBS and concentrated to an OD_600_ = 10. An equal volume of 50% glycerol was added for long-term storage at −80°C. After thawing, the bacteria were concentrated by centrifugation and re-suspended in PBS to an OD_600_ = 10.

### BSA labeling

BSA::AlexaFluor^647^ (Invitrogen, Carlsbad, CA) was resuspended in PBS then added to liquid NGM cooled to 55°C at a final concentration of 1 mg/mL immediately prior to pouring the agar plates. These agar plates were then seeded with OP50, and ∼50 transgenic L4 stage animals were transferred onto the plates and incubated overnight at 22°C in the dark. The following day, labeled animals were transferred onto regular NGM plates seeded with OP50 for 4 hours prior to confocal imaging.

### RNAi

Vector inserts of each RNAi clone were verified by DNA sequencing. RNAi was prepared as previously described [46]. Briefly, an overnight culture of HT115 bacteria containing the RNAi plasmid, was diluted 1 in 50 with LB ampicillin (100 µg/ml) and IPTG (1.5 mm) and grown with shaking at 37°C to an OD_600_ = 0.5. The cultures were concentrated in 3 ml LB ampicillin broth and used to seed NGM plates containing 1.5 mm IPTG and 100 µg/ml ampicillin. Of note, the *cdc-48(RNAi)* and *npl-4(RNAi)* treatments contained a combination of both *cdc48.1* and *cdc-48.2* and *npl4.1* and *npl4.2* RNAi cultures, respectively, as previous studies showed that knockdown of both genes was required to affect ERAD [24]. In these studies, approximately 60–100 L4 stage animals were grown on RNAi plates. For ERAD and autophagy RNAi panels, RNAi treatment was carried out for 48 h with animals being transferred to fresh RNAi plates after 24 h. For proteasomal RNAi studies, RNAi treatment was carried out for 24 h.

### Proteasomal inhibitor plate preparation

Proteasome inhibitor containing NGM plates were prepared by adding 3 µl Bortezomib (50 mm in DMSO, LC Laboratories, Woburn, MA) or 1.2 µl MG132 (50 mm in DMSO, Sigma-Aldrich, St. Louis, MO) directly to 6 ml NGM media cooled to 50°C at to reach a final concentration of 25 µm and 10 µm, respectively, prior to seeding with OP50. L4 stage animals (n = 60–100) were added onto individual drug or control (0.05% DMSO only) plates. Animals remained on plates for 18–24 h at 22°C before analysis.

### Animal sorting using the COPAS™ BIOSORT

Animals were sorted using the COPAS™ BIOSORT (Union Biometrica, Holliston, MA, USA) as previously described [21]. Briefly, transgenic *C. elegans* lines were washed from NGM plates, allowed to settle by gravity and washed several times in PBS to remove large agar particles. The final pellet was resuspended in approximately 40 ml PBS+0.01% Triton X-100 volume to give a flow rate of 10–20 animals per second through the flow cell. L4 stage worms were selected using empirically determined size, as measured by time-of-flight (TOF) through the flow cell, and coefficient of extinction values, determined using the 488 nm solid state laser and an extinction photodiode. Sorted L4 animals were additionally gated based upon the co-injection fluorophore intensity (GFP, YFP or mCherry) using the same 488 nm solid state laser and photomultiplier tubes. Gating and sorting parameters were controlled *via* the COPAS software.

### Imaging of transgenic animals using ArrayScan V^TI^

Imaging using the ArrayScan V^Ti^ (Cellomics, Thermo Fisher, Pittsburgh, PA, USA) was performed as previously described in [21]. Briefly, 30–35 adult stage worms were transferred to a well of a 384-well optical bottom plate (Nunc, Thermo Fisher, Rochester, NY, USA) in 100 µl of PBS, 0.01% Triton X100 and 50 mm sodium azide (NaN_3_). Sodium azide was used as an anesthetizing reagent to ensure the animals were motionless and in a uniform plane. Images were acquired with the automated ArrayScan V^TI^ HCS Reader (Cellomics, ThermoFisher, Pittsburgh, PA), which consists of a Carl Zeiss 200M inverted microscope fitted with a 5× objective and a 0.63× coupler; an LED light engine with excitation wavelengths of 485 nm and 549 nm for green and red fluorophores, respectively; a Hamamatsu ORCA-ER CCD camera; and ArrayScan BioApplications software to simultaneously acquire and analyze images. To acquire images, the autofocus parameters were set to the head marker, either the TRITC channel (Em 590 nm) for P*_myo-2_*mCherry or the FITC channel (Em 535 nm) for P*_myo-2_*GFP containing strains. After autofocusing, the ArrayScan software was programmed to capture images in both FITC and TRITC channels. In order for the ArrayScan V^Ti^ to distinguish between the head and the intestine, worm strains with P*_myo-2_*mCherry expressed GFP or YFP containing proteins in the intestine, and P*_myo-2_*GFP containing strains expressed mCherry. The ArrayScan V^Ti^ SpotDetector BioApplication simultaneously analyzed the fluorescence in both channels above an empirically determined threshold, counting both P*_myo-2_* head marker (number of worms) and intestinal fluorescence. To determine the threshold, several wells with both high and low transgene expression levels were manually selected to ensure proper focus, camera exposure and spot detection threshold settings. For those transgenic worms exhibiting discreet ER accumulations within the intestine (*e.g.* P*_nhx-2_* CPL-1^W32A;Y35A^::YFP), the threshold was higher to determine those objects (spots) above background intestinal autofluorescence, and the spot total area was determined across the entire well. For transgenic lines that exhibited diffuse cytoplasmic expression across the entire intestine (e.g. P*_hsp-4_*GFP), the threshold was lowered to detect the entire intestine, and spot total intensity was used to compare transgene expression in different animals. In order to normalize for the number of animals detected in each well, the spot total area or spot total intensity parameters were divided by the number of head objects detected in the head marker channel.

### Statistical analysis

All studies were repeated for at least three trials (n = 3) unless otherwise noted. With the exception of the proteasomal studies, for each experimental trial, the data was normalized to the baseline controls (*vector(RNAi)* or N2 genetic background) and represented as 1. For these treatments, the data was expressed as a fold-change from *vector* control. Due to increased variability in the proteasomal studies, the data was expressed as total fluorescence intensity per animal. For these assays, animals for each treatment were transferred from three identically treated plates to three wells in a 384 well plate. Approximately 40–60 animals were cultured per plate. For each of the three wells, spot total intensity was divided by the number of heads to provide spot total intensity per animal. This process was repeated two additional times, which provided nine spot total intensity/animal measurements (n = 9). Graphs were reported as an average of the trials and the standard deviation was calculated and plotted to generate error bars. The experiments were tested for statistical significance as compared to control animals using an unpaired, 2-tailed, unequal variance t-test (p<0.05).

### Microscopic imaging

To prepare animals for imaging, 6 µl of 50 mm NaN_3_ in PBS was transferred to the middle of a 35 mm coverglass bottom dish (MatTek, Ashland, MA). Five to fifteen adult stage animals were transferred to the sodium azide solution and covered with a 12 mm circular coverslip.

Confocal images were taken with a Nikon LiveScan SFC confocal microscope with 488, 561 and 647 nm excitation lasers using a 60× 1.4 NA oil Apochromat objective over a 20 micron Z-range at 0.6 micron Z-step and a pinhole size of 60 micrometers. Images were displayed as 2D maximum intensity projections, rendered and analyzed using NIS Elements software.

Widefield images were collected using an automated Nikon Ti Eclipse microscope with a single Z-plane using a 20× objective and ASI motorized stage. Epifluorescence was captured using FITC (Ex 488 nm; Em 512 nm) and TRITC (Ex 568 nm, Em 620 nm) filter sets (Chroma). Images were captured using a Coolsnap HQ2 CCD camera (Photometrics). Exposure settings remained constant throughout the image capture and between transgenic strains. Images were rendered and analyzed using NIS Elements software.

### Lifespan analysis

Lifespan analysis studies were performed at 20°C as previously described [47]. Briefly, 25 L4 stage transgenic animals (N2 Bristol, CPL-1::YFP, and CPL-1^W32A;Y35A^:YFP) were added to NGM plates seeded with OP50 (day 0), and were transferred daily onto new seeded plates for the duration of adult life. The total number of observations equals the number of animals that were scored as dead plus the total number of animals that were censored in the experimental group because they had moved off the plate or were bagged. In these studies, p values were calculated using the log-rank test in the Prism® (GraphPad Software, La Jolla, CA, USA) statistical software package.

### Immunoblotting

#### Denaturing Conditions

For each strain used, ∼500 adult animals were collected into a 1.5 ml microfuge tube containing 0.5 ml PBS. Animals were washed with PBS a total of 3 times to remove any remaining bacteria. Animals were pelleted using a tabletop microfuge and the supernatant was removed. Animals were solubilized by adding 100 µl of 5X Laemmli Buffer containing 0.1% SDS and 5% β-mercaptoethanol to each pellet. Animal pellets in sample buffer were then subjected to 3 bursts of sonication for 10 s each and were boiled for 10 min. Samples were then loaded onto a Criterion™ TGX Any KD precast gel (Bio-Rad, Hercules, CA, USA). Electrophoresis and transfer to nitrocellulose (Bio-Rad) was performed using the Criterion™ Western blotting system (Bio-Rad). Membranes were then incubated for 2 hours with anti-GFP polyclonal antibody (Sigma), or anti-tubulin monoclonal antibody (Sigma). Membranes were then washed for a total of 1 h in TBS+0.01% (v/v) Triton-X 100 before they were incubated for 1 h in either bovine anti-rabbit- or bovine anti-mouse-HRP (Santa Cruz Biotechnology, Santa Cruz, CA, USA) conjugated secondary antibody. HRP-reactive bands were detected using Super Signal® West Pico chemiluminescent substrate (Thermo Fisher Scientific, Pierce, Rockford, IL, USA), and membranes were exposed to Blue Ultra autorad film (ISC BioExpress, Kaysville, UT, USA). The relative molecular mass of immunoreactive bands was assessed using Precision Plus Protein Standards (Bio-Rad). Membranes were stripped in Western Stripping Buffer containing 750 mm glycine (pH 2.0), 1% Tween 20 and 0.1% SDS.

#### Native Conditions

Immunoblotting procedure was carried out as described above with slight modification. Animal pellets were resuspended in 100 µl of PBS containing a protease inhibitor cocktail (Complete mini-tablets; Roche, Indianapolis, IN, USA). Samples were then briefly sonicated for 3 s bursts on ice. This was repeated until breakdown of worm cuticle was observed. Following sonication, samples were centrifuged as above and the supernatant was transferred to a new tube. Five microliters of undiluted sample combined with 20 µl of Native sample buffer (Bio-Rad) were loaded onto a Criterion™ TGX Any KD precast gel (Bio-Rad). Electrophoresis was performed using Tris/Glycine buffer containing 25 mM Tris and 192 mM Glycine.

### Semi-quantitative RT-PCR

Approximately 350–500 transgenic animals were collected in PBS and pelleted from each of the RNAi plates. Pellets were then resuspended in 100 µl of PBS and 400 µl of Trizol reagent (Invitrogen). Animals were lysed by freeze thaw, an additional 200 µl of Trizol reagent and vigorous shaking at 22°C. After the addition of 140 µl of chloroform, samples were centrifuged at 12,000× *g* at 4°C for 15 minutes. The aqueous phase was removed and transferred to a new RNAse free 1.5 ml microfuge tube and an equal volume of 70% ethanol was added. Total RNA was isolated using the RNAeasy kit (Qiagen, Valencia, CA) according to supplier's specifications. The cDNA was made using the SuperScript® III first strand synthesis supermix for qRT-PCR kit (Invitrogen) along with no reverse transcriptase negative controls. CPL-1, HRD-1, HRDL-1 and AMA-1 cDNAs were amplified using the Phusion® high fidelity PCR kit (NEB) from a 10-fold serial dilution of cDNA (from 1∶1 to 1∶10,000) for each given RNAi treatment. Primer sets 15, 16, 17 and 18 were used to amplify HRD-1, HRDL-1, AMA-1 and CPL-1, respectively.

## Supporting Information

Figure S1 **A yeast carboxypeptidase Y/cathepsin A-like fusion gene, F13D12.6::YFP did not traffic to the endo-lysosomal compartment and its mutant did not accumulate in the ER.** (A) Amino acid alignment of yeast CPY [EDV11788.1], human cathepsin A [CAI20250.1], and six *C. elegans* homologues (identified by BLAST) using the ClustalW algorithm. Accession numbers for aligned potential *C. elegans* CPY homologues are as follows: F13D12.6 [CAA88947.1], C08H9.1 [CAA91143.1], K10B2.2a [CCD66392.1], F41C3.5 [CCD65861.1], F32A5.3 [CCD66273.1], and Y40D12A.2 [CCD64385.1]. Blue shading/highlighting is pre-pro domain, the green box is the excision peptide and red boxes are catalytic triad residues. (B–I) Transgenic animals expressing F13D12::YFP (B–E) or F13D12.6^G166R^::YFP (F–I) were examined by confocal microscopy. Both lines were co-injected with a DsRed::KDEL transgene to mark the ER (C, G), and were incubated with BSA::AlexaFluor^647^ to label the endo-lysosomal compartment (D, H). Both F13D12.6::YFP and F13D12.6^G166R^::YFP demonstrated a fine reticular pattern within intestinal cells that co-localized with DsRed::KDEL (E, I). The wild-type protein did not co-localize with BSA::AlexaFluor^647^, suggesting that this protein did not traffic to the endo-lysosomal compartment. Scale bar represents 10 µm. (J) Immunoblot of protein lysates from two different transgenic strains expressing F13D12.6^G166R^::YFP. Blot probed with anti-GFP/YFP antisera. *M*_r_ fusion protein = 77-kDa. (K) The mutant protein, CPL-1^W32A;Y35A^::YFP, but not F13D12.6^G166R^::YFP, accumulated in the ER after ERAD knockdown using *cdc-48(RNAi)*, *hrd-1(RNAi)* or *sel-2(RNAi)*; suggesting that the latter mutant protein was not an ERAD substrate under these experimental conditions.
(TIF)

Figure S2 **Expression of CPL-1^W32A;Y35A^::YFP does not affect** ***C. elegans*** **lifespan.** Kaplan-Meier survival curves were generated for N2 (blue), P*_nhx-2_cpl-1::YFP* (green), or P*_nhx-2_cpl-1* ^W32A;Y35A^::YFP (red) animals to determine if animal longevity was affected by expression of either transgene. Individual strains had mean survival times of 300 h (N2), 360 h (CPL-1::YFP), and 312 h (CPL-1^W32A;Y35A^::YFP). Statistical significance compared to control was assessed by log-rank test, which indicated no statistical difference between strains (N2 vs. CPL-1::YFP, *p* = 0.4; N2 vs. CPL-1^W32A;Y35A^, *p* = 0.7). Assays were performed at 20°C and the total number of observations counted were equal to the number of animals that died plus the number of censored animals.
(TIF)

Figure S3 **Controls for ERAD RNAi effectiveness.** (A) P*_nhx-2_*YFP or (B) P*_hsp-4_*GFP animals were exposed to the ERAD RNAi panel for 48 h and processed as described in Figure 3. The algorithm was adjusted to detect the entire intestinal fluorescence pattern above that of the *vector(RNAi)* control. Total intensity per animal was used in place of total area. Statistical analysis of the RNAi treated animals relative to vector was performed using an unpaired, 2-tailed *t*-test (unequal variance) (*p<0.05). No statistical difference in total YFP fluorescence was observed for all tested RNAi's, suggesting RNAi treatment did not alter levels of transgene expression by activating the *nhx-2* promoter (A). All RNAi's tested significantly raised P*_hsp-4_*GFP expression levels except for *hrdl-1(RNAi)* as previously described [13], indicating the UPR activation by RNAi treatment (B). (C) Effectiveness of *hrdl-1(RNAi)* was demonstrated by showing knockdown of steady-state HRDL-1 mRNA levels by semi-quantitative RT-PCR. Total RNA was isolated from CPL-1^W32A;Y35A^ animals treated with either *vector*, *hrd-1*, or *hrdl-1* RNAi. RT-PCR (+/−RT) reactions were performed on a 10-fold serial dilution of cDNA's from each RNAi condition to amplify HRD-1, HRDL-1, or AMA-1. HRDL-1 cDNA was not detected after the *hrdl-1(RNAi)*. AMA-1 and genomic (g) DNA served as RT and amplification controls, respectively.
(TIF)

Figure S4 **Autophagy RNAi controls.** mCherry::LGG-1 expressing animals were exposed to an autophagy RNAi panel for 48 hours and starved for 4 hours to induce autophagy. Images of 5–10 animals were collected using a widefield epifluorescence microscopy. In *vector(RNAi)* animals the mCherry::LGG-1 expression profile shifted from a diffuse to a more punctate distribution, indicating autophagosome formation, while treatment with *lgg-1(RNAi)* successfully reduced the mCherry::LGG-1 fluorescence to below detectable levels. *unc-51(RNAi)* or *bec-1(RNAi)* also prevented puncta formation, indicating suppression of autophagosome formation.
(TIF)

Table S1 **Transgene List.**
(DOCX)

Table S2 **PCR primer pairs for transgene construction.**
(DOCX)

Table S3 ***C. elegans*** **strain list.**
(DOCX)
