# Supplementary figures and images for: A Pro-Cathepsin L Mutant Is a Luminal Substrate for Endoplasmic-Reticulum-Associated Degradation in C. elegans
Source: PLoS One. 2012 Jul 2;7(7):e40145. doi: 10.1371/journal.pone.0040145 (PMC3388072; doi:10.1371/journal.pone.0040145)

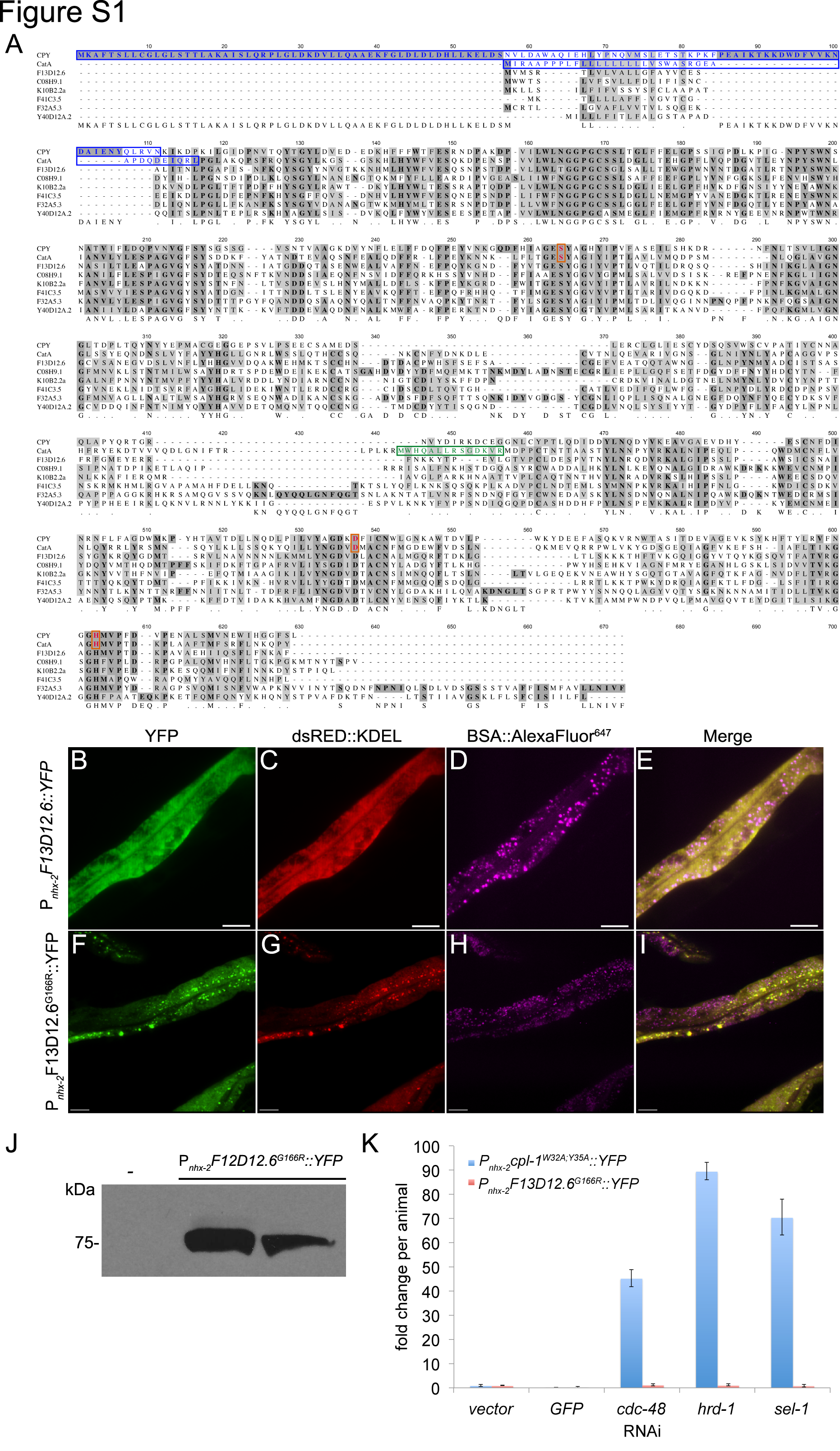

Supplement: Figure S1 — A yeast carboxypeptidase Y/cathepsin A-like fusion gene, F13D12.6::YFP did not traffic to the endo-lysosomal compartment and its mutant did not accumulate in the ER. (A) Amino acid alignment of yeast CPY [EDV11788.1], human cathepsin A [CAI20250.1], and six C. elegans homologues (identified by BLAST) using the ClustalW algorithm. Accession numbers for aligned potential C. elegans CPY homologues are as follows: F13D12.6 [CAA88947.1], C08H9.1 [CAA91143.1], K10B2.2a [CCD66392.1], F41C3.5 [CCD65861.1], F32A5.3 [CCD66273.1], and Y40D12A.2 [CCD64385.1]. Blue shading/highlighting is pre-pro domain, the green box is the excision peptide and red boxes are catalytic triad residues. (B–I) Transgenic animals expressing F13D12::YFP (B–E) or F13D12.6G166R::YFP (F–I) were examined by confocal microscopy. Both lines were co-injected with a DsRed::KDEL transgene to mark the ER (C, G), and were incubated with BSA::AlexaFluor647 to label the endo-lysosomal compartment (D, H). Both F13D12.6::YFP and F13D12.6G166R::YFP demonstrated a fine reticular pattern within intestinal cells that co-localized with DsRed::KDEL (E, I). The wild-type protein did not co-localize with BSA::AlexaFluor647, suggesting that this protein did not traffic to the endo-lysosomal compartment. Scale bar represents 10 µm. (J) Immunoblot of protein lysates from two different transgenic strains expressing F13D12.6G166R::YFP. Blot probed with anti-GFP/YFP antisera. M r fusion protein = 77-kDa. (K) The mutant protein, CPL-1W32A;Y35A::YFP, but not F13D12.6G166R::YFP, accumulated in the ER after ERAD knockdown using cdc-48(RNAi), hrd-1(RNAi) or sel-2(RNAi); suggesting that the latter mutant protein was not an ERAD substrate under these experimental conditions. (TIF) [file pone.0040145.s001.tif]

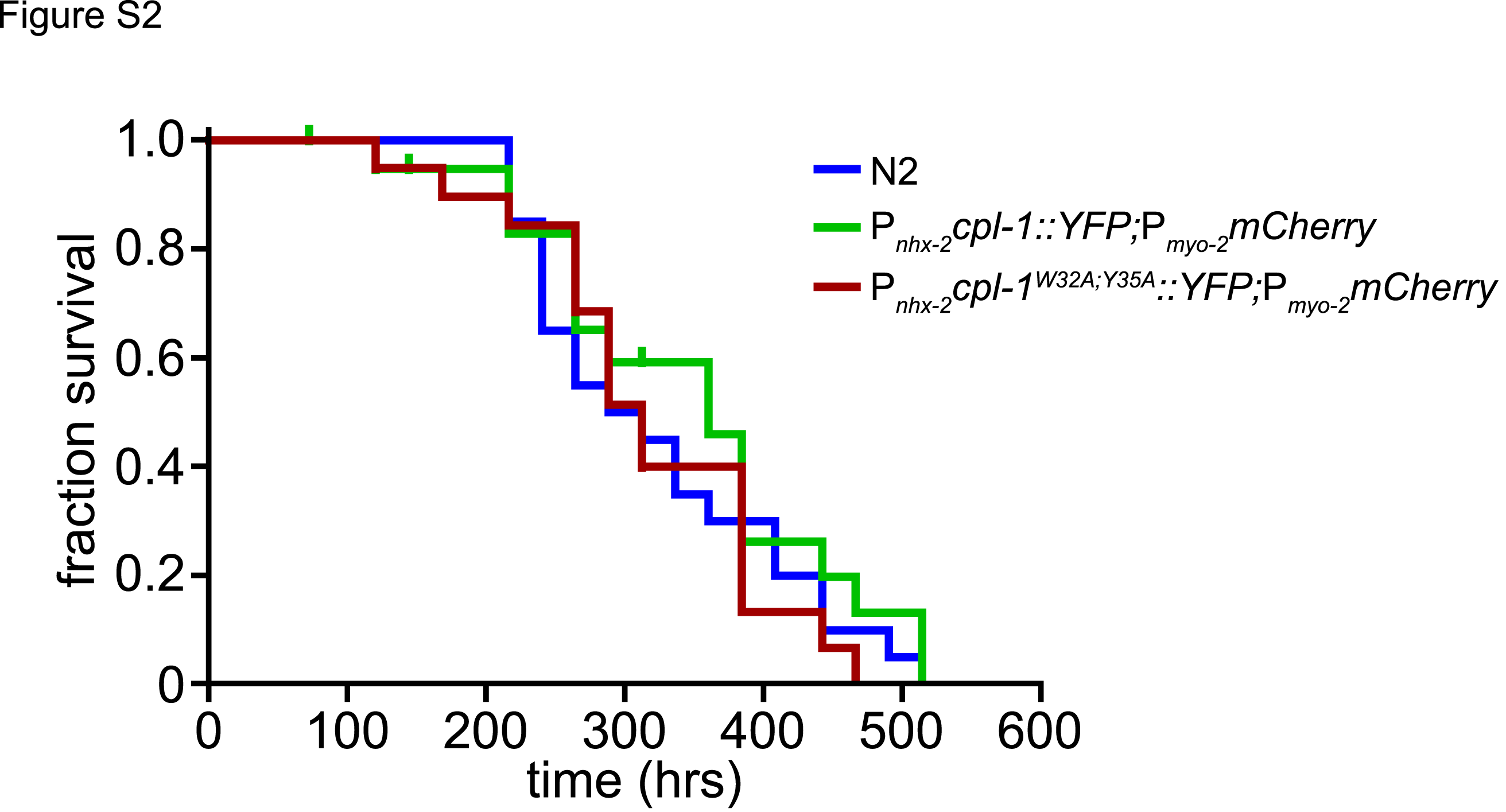

Supplement: Figure S2 — Expression of CPL-1W32A;Y35A::YFP does not affect C. elegans lifespan. Kaplan-Meier survival curves were generated for N2 (blue), Pnhx-2cpl-1::YFP (green), or Pnhx-2cpl-1 W32A;Y35A::YFP (red) animals to determine if animal longevity was affected by expression of either transgene. Individual strains had mean survival times of 300 h (N2), 360 h (CPL-1::YFP), and 312 h (CPL-1W32A;Y35A::YFP). Statistical significance compared to control was assessed by log-rank test, which indicated no statistical difference between strains (N2 vs. CPL-1::YFP, p = 0.4; N2 vs. CPL-1W32A;Y35A, p = 0.7). Assays were performed at 20°C and the total number of observations counted were equal to the number of animals that died plus the number of censored animals. (TIF) [file pone.0040145.s002.tif]

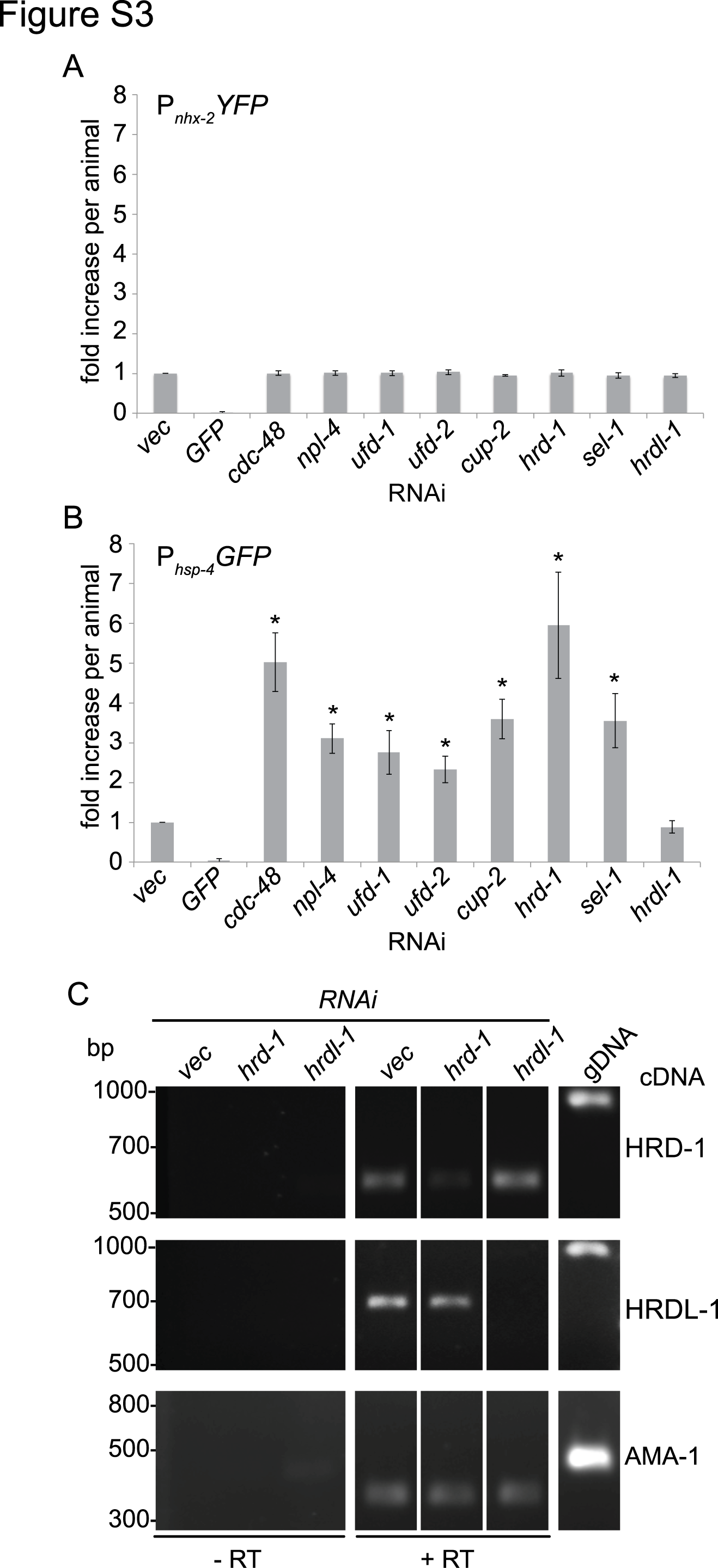

Supplement: Figure S3 — Controls for ERAD RNAi effectiveness. (A) Pnhx-2YFP or (B) Phsp-4GFP animals were exposed to the ERAD RNAi panel for 48 h and processed as described in Figure 3. The algorithm was adjusted to detect the entire intestinal fluorescence pattern above that of the vector(RNAi) control. Total intensity per animal was used in place of total area. Statistical analysis of the RNAi treated animals relative to vector was performed using an unpaired, 2-tailed t-test (unequal variance) (*p<0.05). No statistical difference in total YFP fluorescence was observed for all tested RNAi's, suggesting RNAi treatment did not alter levels of transgene expression by activating the nhx-2 promoter (A). All RNAi's tested significantly raised Phsp-4GFP expression levels except for hrdl-1(RNAi) as previously described [13], indicating the UPR activation by RNAi treatment (B). (C) Effectiveness of hrdl-1(RNAi) was demonstrated by showing knockdown of steady-state HRDL-1 mRNA levels by semi-quantitative RT-PCR. Total RNA was isolated from CPL-1W32A;Y35A animals treated with either vector, hrd-1, or hrdl-1 RNAi. RT-PCR (+/−RT) reactions were performed on a 10-fold serial dilution of cDNA's from each RNAi condition to amplify HRD-1, HRDL-1, or AMA-1. HRDL-1 cDNA was not detected after the hrdl-1(RNAi). AMA-1 and genomic (g) DNA served as RT and amplification controls, respectively. (TIF) [file pone.0040145.s003.tif]

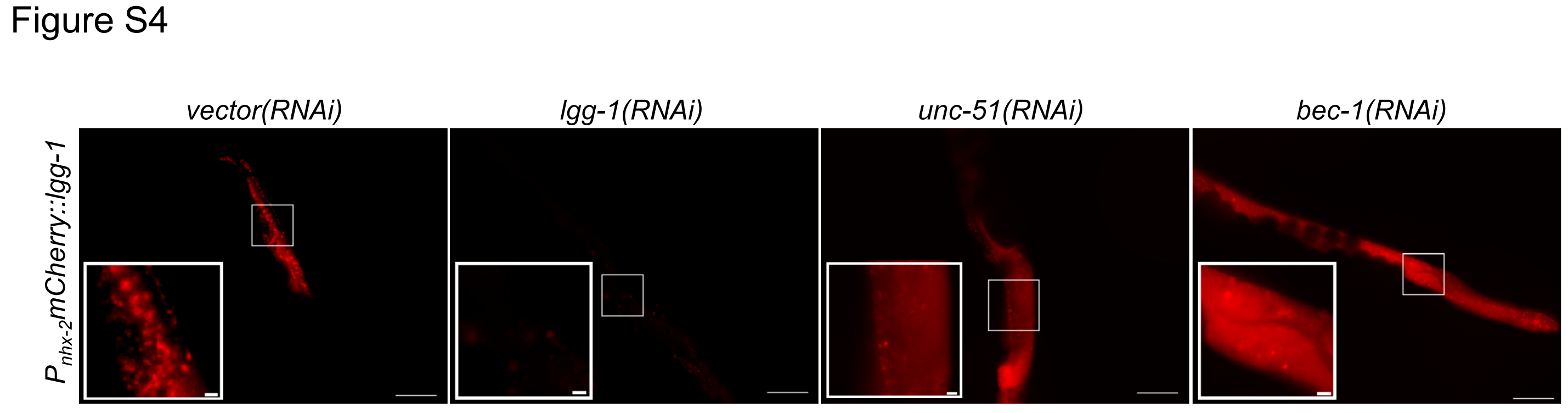

Supplement: Figure S4 — Autophagy RNAi controls. mCherry::LGG-1 expressing animals were exposed to an autophagy RNAi panel for 48 hours and starved for 4 hours to induce autophagy. Images of 5–10 animals were collected using a widefield epifluorescence microscopy. In vector(RNAi) animals the mCherry::LGG-1 expression profile shifted from a diffuse to a more punctate distribution, indicating autophagosome formation, while treatment with lgg-1(RNAi) successfully reduced the mCherry::LGG-1 fluorescence to below detectable levels. unc-51(RNAi) or bec-1(RNAi) also prevented puncta formation, indicating suppression of autophagosome formation. (TIF) [file pone.0040145.s004.tif]
