# Supplementary material for: A Pro-Cathepsin L Mutant Is a Luminal Substrate for Endoplasmic-Reticulum-Associated Degradation in C. elegans
Source: PLoS One. 2012 Jul 2;7(7):e40145. doi: 10.1371/journal.pone.0040145 (PMC3388072; doi:10.1371/journal.pone.0040145)
Supplement: Table S1 — Transgene List. (DOCX) [file pone.0040145.s005.docx]

| **Table S1. Transgene List** | | |
| --- | --- | --- |
| **Plasmid** | **Transgene** | **Vector backbone**  **(all confer ampicillin resistance)** |
| pAV1944 | P*_myo-2_mCherry* | pPD49.26 |
| pAV1997 | *mCherry* | pPD49.26 |
| pAV1951 | P*_nhx-2_mCherry* | pPD49.26 |
| pKS2236 | P*_nhx-2_YFP* | pPD49.26 |
| pKS2301 | P*_nhx-2_cpl-1::YFP* | pPD49.26 |
| pKS2311 | P*_nhx-2_cpl-1^W32A;Y35A^::YFP* | pPD49.26 |
| pNG2462 | P*_nhx-2_F13D12.6::YFP* | pPD49.26 |
| pNG2470 | P*_nhx-2_F13D12.6^G166R^::YFP* | pPD49.26 |
| pAV1825 | P*_nhx-2_DsRed::KDEL* | pPD95.85 |
| pSG2142 | P*_nhx-2_UB-V::mCherry* | pPD49.26 |
| pSG2143 | P*_nhx-2_UB-R::mCherry* | pPD49.26 |
| pSG2144 | P*_nhx-2_UB-M::mCherry* | pPD49.26 |
| pAV2021 | P*_hsp-4_GFP* | pPD95.77 |
