# Supplementary material for: A Pro-Cathepsin L Mutant Is a Luminal Substrate for Endoplasmic-Reticulum-Associated Degradation in C. elegans
Source: PLoS One. 2012 Jul 2;7(7):e40145. doi: 10.1371/journal.pone.0040145 (PMC3388072; doi:10.1371/journal.pone.0040145)
Supplement: Table S2 — PCR primer pairs for transgene construction. (DOCX) [file pone.0040145.s006.docx]

| **Table S2. PCR primer pairs for transgene construction** | | | | |
| --- | --- | --- | --- | --- |
| **Primer Set** | **Forward**  **/Reverse** | **Sequence (5’-3’, restriction site underlined)** | **Restriction**  **site** | **Amplicon size (bp)** |
| 1 | F | TATTATGCTAGCATGGTGAGCAAGGGCGAGGAG | *Nhe*I | 717 |
| 1 | R | ATATAAGATATCCTACTTGTACAGCTCGTCCATGCC | *EcoR*V |  |
| 2 | F | TAATTGCATGCTGGTGGACAGTAACTGTCTG | *Sph*I | 1,147 |
| 2 | R | TATTATCTAGAGAGGGTTAAAATGAAAAGTGGTGG | *Xba*I |  |
| 3 | F | ATGCAGCTAGCATGAGTAAAGGAGAAGAACTTTTCACTGGAG | *Nhe*I | 868 |
| 3 | R | GTATAGAGCTCCTATTTGTATAGTTCATCCATGCCATGTG | *Sac*I |  |
| 4 | F | GAGCAGCTAGCAGAATGAACCGATTCATTCTTCTGGC | *Nhe*I | 2,626 |
| 4 | R | TGCTCGCTAGCGACCAATGGATAACTGGCCTTGGTGGCG | *Nhe*I |  |
| 5 | F | CGAGTCGGCCATCGAGAAA**GCT**GACGAC**GC**TAAGGAGGACTTTGATAAGG |  |  |
| 5 | R | CCTTATCAAAGTCCTCCTTA**GC**GTCGTC**AGC**TTTCTCGATGGCCGACTCG |  |  |
| 6 | F | GGATCCGCTAGCATGGTCATGAGCAGGACACTTG | *Nhe*I | 3,224 |
| 6 | R | AATATAGCTAGCGAAGGCCTTGTTGAACAGG | *Nhe*I |  |
| 7 | F | CGATTTCTATGTGACT**C**GAGAGGGGTACGGTGGAATCTATG |  |  |
| 7 | R | CATAGATTCCACCGTACCCCTCTC**G**AGTCACATAGAAATCG |  |  |
| 8 | F | ATATATAAGCTTGATTCATACTGAAGTAGGTGCC | *Hind*III | 2,033 |
| 8 | R | ATATATTCTAGACGGAGAAGAGACATGATTTAATCAC | *Xba*I |  |
| 9 | F | GGGATTACACATGGCATGGATGAACTATACAAA**GGCGCC**TCGTAGAATTCCAACTGAGCGCCG | *Kas*I |  |
| 9 | R | CGGCGCTCAGTTGGAATTCTACGA**GGCGCC**TTTGTATAGTTCATCCATGCCATGTGTAATCCC | *Kas*I |  |
| 10 | F | TAGGTACCGATGGCCTCCTCCGAGGACGTCATC | *Kpn*I | 675 |
| 10 | R | TAGGCGCCCTA*CAGCTCGTCCTT*CTTGTACAGGAACAGGTGGTGGCGGCC | *Kas*I |  |
| 11 | F | AATTATGCTAGCATGCAGATCTTCGTGAAGACCC | *Nhe*I | 248 |
| 11 | R | AATTATGCTAGC*CTTCAGGTGTCG*ACCAAGCTTCCCCACCACACC | *Nhe*I |  |
| 12 | F | GGTCTTCCGTCTCAGAGGTG**GCCGC**GGGAAGCTTGGTCGACACC |  |  |
| 12 | R | GGTGTCGACCAAGCTTCCC**GCGGC**CACCTCTGAGACGGAAGACC |  |  |
| 13 | F | GGTCTTCCGTCTCAGAGGTGGC**ATG**GGGAAGCTTGGTCGACACC |  |  |
| 13 | R | GGTGTCGACCAAGCTTCCC**CAT**GCCACCTCTGAGACGGAAGACC |  |  |
| 14 | F | TTAATAAAGCTTGAAGTTAAACTCTTCACATTTC | *Hind*III | 1,151 |
| 14 | R | TATTAATCTAGAATTTTTCTCTTGATGAGCGG | *Xba*I |  |
| 15 | F | GCCGATTCGTATTTCGTGTCAAG |  | 598 |
| 15 | R | TCAGCCGCATTTCCTCCTG |  |  |
| 16 | F | CAACTTTTGGCATTCTCACCTGG |  | 703 |
| 16 | R | GGTTCTTGGACTATTTCGGCG |  |  |
| 17 | F | CAGTGGCTCATGTCGAGT |  | 425 |
| 17 | R | CGACCTTCTTTCCATCAT |  |  |
| 18 | F | AAGGACAACACGCTCGCAAG |  | 514 |
| 18 | R | TTACGCCTAACGGTAAGCCTACGC |  |  |

| **Table S3. *C. elegans* strain list** | | |
| --- | --- | --- |
| **Strain Name** | **Genotype** | **Protein expressed** |
| VK689 | *vkIs689[*P*_nhx-2_sGFP::ATM;*P*_myo-2_mCherry]* | sGFP::ATM |
| VK737 | *vkEx737[*P*_hsp-4_GFP;*P*_myo-2_mCherry]* | GFP |
| VK1104 | *vkEx1104[*P*_nhx-2_YFP;*P*_myo-2_mCherry]* | YFP |
| VK1241 | *vkEx1241[*P*_nhx-2_mCherry::lgg-1;*P*_myo-2_GFP]* | mCherry::LGG-1 |
| VK1243 | *vkEx1243[*P*_nhx-2_UB-V::mCherry;*P*_myo-2_GFP]* | UB-V::mCherry |
| VK1244 | *vkEx1244[*P*_nhx-2_UB-M::mCherry;*P*_myo-2_GFP]* | UB-M::mCherry |
| VK1256 | *vkEx1256[*P*_nhx-2_cpl-1::YFP;*P*_nhx-2_DsRed::KDEL]* | CPL-1::YFP |
| VK1258 | *vkEx1258[*P*_nhx-2_cpl-1^W32AY35A^::YFP;*P*_nhx-2_DsRed::KDEL]* | CPL-1^W32AY35A^::YFP |
| VK1260 | *vkEx1260[*P*_nhx-2_cpl-1::YFP;*P*_myo-2_mCherry]* | CPL-1::YFP |
| VK1770 | *vkEx1770[*P*_nhx-2_F13D12.6::YFP;*P*_nhx-2_DsRed::KDEL]* | F13D12.6::YFP |
| VK1870 | *vkEx1870[*P*_nhx-2_F13D12.6^G166R^::YFP;*P*_myo-2_mCherry]* | F13D12.6^G166R^::YFP |
| VK1879 | *vkEx1879[*P*_nhx-2_cpl-1^W32AY35A^::YFP;*P*_myo-2_mCherry]* | CPL-1^W32AY35A^::YFP |
| VK1984 | *unc-51(e369);vkEx1879[*P*_nhx-2_cpl-1^W32AY35A^::YFP;*P*_myo-2_mCherry];line 3* | CPL-1^W32AY35A^::YFP |
| VK1985 | *unc-51(e369);vkEx1879[*P*_nhx-2_cpl-1^W32AY35A^::YFP;*P*_myo-2_mCherry];line 4* | CPL-1^W32AY35A^::YFP |
