# Supplementary material for: A Pro-Cathepsin L Mutant Is a Luminal Substrate for Endoplasmic-Reticulum-Associated Degradation in C. elegans
Source: PLoS One. 2012 Jul 2;7(7):e40145. doi: 10.1371/journal.pone.0040145 (PMC3388072; doi:10.1371/journal.pone.0040145)
Supplement: Table S3 — C. elegans strain list. (DOCX) [file pone.0040145.s007.docx]

| **Table S3. *C. elegans* strain list** | | |
| --- | --- | --- |
| **Strain Name** | **Genotype** | **Protein expressed** |
| VK689 | *vkIs689[*P*_nhx-2_sGFP::ATM;*P*_myo-2_mCherry]* | sGFP::ATM |
| VK737 | *vkEx737[*P*_hsp-4_GFP;*P*_myo-2_mCherry]* | GFP |
| VK1104 | *vkEx1104[*P*_nhx-2_YFP;*P*_myo-2_mCherry]* | YFP |
| VK1241 | *vkEx1241[*P*_nhx-2_mCherry::lgg-1;*P*_myo-2_GFP]* | mCherry::LGG-1 |
| VK1243 | *vkEx1243[*P*_nhx-2_UB-V::mCherry;*P*_myo-2_GFP]* | UB-V::mCherry |
| VK1244 | *vkEx1244[*P*_nhx-2_UB-M::mCherry;*P*_myo-2_GFP]* | UB-M::mCherry |
| VK1256 | *vkEx1256[*P*_nhx-2_cpl-1::YFP;*P*_nhx-2_DsRed::KDEL]* | CPL-1::YFP |
| VK1258 | *vkEx1258[*P*_nhx-2_cpl-1^W32AY35A^::YFP;*P*_nhx-2_DsRed::KDEL]* | CPL-1^W32AY35A^::YFP |
| VK1260 | *vkEx1260[*P*_nhx-2_cpl-1::YFP;*P*_myo-2_mCherry]* | CPL-1::YFP |
| VK1770 | *vkEx1770[*P*_nhx-2_F13D12.6::YFP;*P*_nhx-2_DsRed::KDEL]* | F13D12.6::YFP |
| VK1870 | *vkEx1870[*P*_nhx-2_F13D12.6^G166R^::YFP;*P*_myo-2_mCherry]* | F13D12.6^G166R^::YFP |
| VK1879 | *vkEx1879[*P*_nhx-2_cpl-1^W32AY35A^::YFP;*P*_myo-2_mCherry]* | CPL-1^W32AY35A^::YFP |
| VK1984 | *unc-51(e369);vkEx1879[*P*_nhx-2_cpl-1^W32AY35A^::YFP;*P*_myo-2_mCherry];line 3* | CPL-1^W32AY35A^::YFP |
| VK1985 | *unc-51(e369);vkEx1879[*P*_nhx-2_cpl-1^W32AY35A^::YFP;*P*_myo-2_mCherry];line 4* | CPL-1^W32AY35A^::YFP |
